# Controversies and progress on standardization of large-scale brain network nomenclature

**DOI:** 10.1162/netn_a_00323

**Published:** 2023-10-01

**Authors:** Lucina Q. Uddin, Richard F. Betzel, Jessica R. Cohen, Jessica S. Damoiseaux, Felipe De Brigard, Simon B. Eickhoff, Alex Fornito, Caterina Gratton, Evan M. Gordon, Angela R. Laird, Linda Larson-Prior, A. Randal McIntosh, Lisa D. Nickerson, Luiz Pessoa, Ana Luísa Pinho, Russell A. Poldrack, Adeel Razi, Sepideh Sadaghiani, James M. Shine, Anastasia Yendiki, B. T. Thomas Yeo, R. Nathan Spreng

**Affiliations:** Department of Psychiatry and Biobehavioral Sciences and Department of Psychology, University of California, Los Angeles, Los Angeles, CA, USA; Department of Psychological and Brain Sciences, Indiana University, Bloomington, IN, USA; Department of Psychology and Neuroscience, University of North Carolina, Chapel Hill, NC, USA; Institute of Gerontology and Department of Psychology, Wayne State University, Detroit, MI, USA; Department of Philosophy, Duke University, Durham, NC, USA; Institute of Systems Neuroscience, Heinrich Heine University Düsseldorf, Düsseldorf, Germany; Turner Institute for Brain and Mental Health, Monash University, Melbourne, Australia; Department of Psychology, Northwestern University, Evanston, IL, USA; Mallinckrodt Institute of Radiology, Washington University, St. Louis, MO, USA; Department of Physics, Florida International University, Miami, FL, USA; Deptartment of Psychiatry and Department of Neurobiology and Developmental Sciences, University of Arkansas for Medical Sciences, Little Rock, AR, USA; Institute for Neuroscience and Neurotechnology, Simon Fraser University, Vancouver, BC, Canada; Department of Psychiatry, McLean Hospital, Boston, MA, USA; Department of Psychology, University of Maryland, College Park, MD, USA; Brain and Mind Institute, Western University, London, Ontario, Canada; Department of Psychology, Stanford University, Stanford, CA, USA; Department of Psychology, University of Illinois, Urbana Champaign, IL, USA; Brain and Mind Center, University of Sydney, Sydney, Australia; Department of Radiology, Massachusetts General Hospital, Boston, MA, USA; Department of Electrical and Computer Engineering, National University of Singapore, Singapore; Department of Neurology and Neurosurgery, McGill University, Montreal, Canada

**Keywords:** Brain network, Cognitive neuroscience, Diffusion weighted imaging, Functional connectivity, Network neuroscience, Parcellation, Resting-state fMRI, Structural connectivity, EEG, MEG

## Abstract

Progress in scientific disciplines is accompanied by standardization of terminology. Network neuroscience, at the level of macroscale organization of the brain, is beginning to confront the challenges associated with developing a taxonomy of its fundamental explanatory constructs. The Workgroup for HArmonized Taxonomy of NETworks (WHATNET) was formed in 2020 as an Organization for Human Brain Mapping (OHBM)–endorsed best practices committee to provide recommendations on points of consensus, identify open questions, and highlight areas of ongoing debate in the service of moving the field toward standardized reporting of network neuroscience results. The committee conducted a survey to catalog current practices in large-scale brain network nomenclature. A few well-known network names (e.g., default mode network) dominated responses to the survey, and a number of illuminating points of disagreement emerged. We summarize survey results and provide initial considerations and recommendations from the workgroup. This perspective piece includes a selective review of challenges to this enterprise, including (1) network scale, resolution, and hierarchies; (2) interindividual variability of networks; (3) dynamics and nonstationarity of networks; (4) consideration of network affiliations of subcortical structures; and (5) consideration of multimodal information. We close with minimal reporting guidelines for the cognitive and network neuroscience communities to adopt.

## DEFINITIONS AND SCOPE: WHAT IS A “LARGE-SCALE BRAIN NETWORK”?

The standardization of terminology and nomenclature is an essential element of progress in any scientific endeavor. In the field of network neuroscience, the most fundamental nomenclature rests on the definition of a brain network itself. Some important definitions in network neuroscience are given as Technical Terms in this article. At the most general level, a network is a system of interacting elements, which can be modeled as a graph of nodes connected by edges. The current Perspective represents a problem statement wherein we discuss a fundamental issue in this nascent field: inconsistencies in large-scale brain network nomenclature. The target audience is new and established researchers in the fields of network and cognitive neuroscience. The ultimate goal of this work is to provide recommendations on points of consensus, identify open questions, and highlight areas of ongoing debate in the service of moving the field toward greater standardization of reporting for network neuroscience results.

In this rapidly evolving field, there is a need to define the scale at which brain networks are being examined. Neuroimaging has focused on the macroscale relationships between brain regions in defining networks and their interactions. This scale emerges in part by virtue of data acquisition parameters, which limit resolution to levels predominantly coarser than a cubic millimeter. This manuscript will focus on networks at the macroscale, using the term ‘large-scale brain network’ to refer to what have variably been called “resting-state networks” (Luca et al., 2006), “intrinsic connectivity networks” (Seeley et al., 2007), or “functional brain networks” (Power et al., 2011a) in the literature describing coherent brain signal fluctuations recorded with noninvasive neuroimaging.

One of the key issues in defining networks is that the very notion has different meanings across scientific domains, and even subfields within cognitive neuroscience and neuroimaging. The term ‘network’ has been used to refer to patterns of coactivation, spatial patterns of correlated signals derived from multivariate analyses (e.g., independent component analysis, ICA), or a distributed pattern of regional brain activation. In network science, however, the term ‘network’ is defined unambiguously. Specifically, it is used to refer to a collection of nodes and edges: nodes represent the fundamental units of a system and edges represent their pairwise interactions. This definition is compatible with some, but not all, of the definitions used in neuroimaging. Namely, it agrees with network or graphical models of the brain, wherein nodes correspond to discrete neural elements (e.g., cells, neuronal populations, brain areas, neuroanatomical regions), and edges correspond to structural, functional, and effective connections among nodes. However, the precise way in which networks are defined is highly variable, and can sometimes lead to inconsistent definitions of specific brain networks.

The field of large-scale network neuroscience is only beginning to confront the challenges associated with developing and refining a taxonomy of relevant entities. At present, the field lacks consensus around basic questions such as, “What constitutes a large-scale brain network?”; “Are there reliable networks that can be observed in the brains of all individuals?”; and, “What reporting conventions should be adopted to facilitate cross-laboratory communication?” One can see that these questions are somewhat philosophical in nature, and are often assumed by researchers to have converged on greater consensus than the literature suggests. Take, for example, the output of a PubMed search for the term ‘default network,’ which includes over 10,000 articles. The term ‘default mode’ of brain function was introduced into the lexicon by Raichle and colleagues in 2001, who used positron emission tomography to measure brain oxygen extraction. They reported that medial prefrontal cortex (MPFC), posterior cingulate cortex (PCC), and lateral parietal and temporal cortices consistently showed decreased activity across a range of cognitive paradigms compared with rest (Raichle et al., 2001; Shulman et al., 1997). Greicius and colleagues used resting-state (Biswal et al., 1995) functional magnetic resonance imaging (fMRI) a few years later to examine functional connectivity among these task deactivated brain regions, conducting the first network analysis of the default mode hypothesis (Greicius et al., 2003). It was this 2003 study that first introduced the term ‘default mode network’, which in subsequent work often has been shortened to “default network” (Buckner et al., 2008). Even a cursory glance at the multitude of topographical depictions of this commonly studied large-scale brain network reveals that, although there is a strong intuition that we “know it when we see it” in functional neuroimaging data, precise definitions of what constitutes the network in terms of critical nodes, affiliated brain regions, estimation of regional interactions, and other quantitative descriptors are often missing from published reports. To further complicate matters, the somewhat common division of the default network into subnetworks (Andrews-Hanna et al., 2010; Damoiseaux et al., 2008; Uddin et al., 2009) illustrates issues surrounding network scale, resolution, and hierarchies—a topic we will return to later in the discussion. These subnetworks (which include some, but not all, nodes of the default network) partially overlap in spatial extent, although the precise separation and extent of spatial overlap depends on the analytic method used. For example, spatial ICA (sICA) at high dimensionality can be used to fractionate the default network into anterior and posterior subsystems (Kiviniemi et al., 2009). In contrast, hierarchical clustering of interregional resting-state correlations dissociates medial temporal lobe and dorsal MPFC subsystems of the default network (Andrews-Hanna et al., 2010). Whether or not the results from fractionating a larger network should be considered subnetworks or proper networks themselves illustrates the complexity of developing standardized network definitions.

Appropriate methods for defining and characterizing large-scale brain networks in neuroimaging data have been discussed and debated for over 25 years (Friston, 1994). Recently, a draft network taxonomy rooted in anatomical terminology was proposed, delineating six canonical networks labeled “occipital,” “pericentral,” “dorsal fronto-parietal,” “lateral fronto-parietal,” “midcingulo-insular,” and “medial fronto-parietal” (Uddin et al., 2019). This focus on anatomy was intended to move away from functional names that are commonly assigned to specific networks (e.g., default, executive control, salience) that may only be appropriate in certain psychological contexts, imply an unnecessarily strong degree of structure-function correspondence, and often lack anatomical precision. The proposal acknowledged that the draft network taxonomy provided an incomplete characterization that should be refined in future iterations through a dedicated working group. Pursuant to this, the Organization for Human Brain Mapping (OHBM) Committee on Best Practices instantiated the Workgroup for HArmonized Taxonomy of NETworks (WHATNET), comprising the current authors. The current manuscript represents this work in progress, providing an update and further considerations for building a consensus network terminology.

One consideration that is germane to this topic is whether we consider one particular neuroimaging modality (e.g., resting-state fMRI, task-based fMRI, diffusion MRI (dMRI), electroencephalography (EEG)) to be the most relevant from which to search for empirical support for a given network taxonomy proposal. After months of deliberation, the WHATNET committee decided that a truly universal taxonomy, if at all plausible, should encompass all relevant findings from multiple neuroimaging modalities, highlighting points of maximal multimodal convergence where appropriate. Still, as the vast majority of the current human connectomics literature has focused on brain networks derived from fMRI data, the findings from this imaging modality are likely to dominate the discussion, with structural and electrophysiological neuroimaging modalities considered as complementary approaches at this time (see section Contributions of Multimodal Information). This balance may shift with future advances in neuroimaging technologies and their adoption in network neuroscience research. In the current work, we take the precedent set by the original Committee on Best Practices in Data Analysis and Sharing (COBIDAS) effort that focused first on fMRI reporting guidelines (Nichols et al., 2017) and later expanded to include EEG and magnetoencephalography (MEG) (Pernet et al., 2020). We discuss considerations for network neuroscience research primarily in the fMRI domain and present initial guidelines for reporting results. We expect that these best practices will be updated and expanded to other imaging modalities in future iterations.

## SUMMARY OF OHBM SURVEY RESULTS AND IMPLICATIONS

In 2021, WHATNET conducted an online survey of the OHBM community to explore current trends in large-scale brain network naming conventions. The objective of this survey was twofold: (1) to identify which terms the neuroscience community is using to characterize and describe large-scale brain networks, and (2) to determine the degree of consensus that already exists in the identification of network topographies. Members of WHATNET provided a range of images depicting large-scale brain networks derived from their own research or other sources to be used in the survey. Survey participants provided information on their academic background, including training and neuroimaging methods experience. For the survey, a randomized sequence of 93 brain network images and 7 “lure” network ensembles were presented, depicting network images in the brain volume and on the cortical surface. Participants were asked to provide open responses to the prompt: “Please name this network.”

A total of 956 individuals enrolled in the survey. Of those, 611 responded to some of the questions, and only a total of 77 completed all of the questions. Of the 611 partial respondents, 46% were members of the OHBM, 1% were undergraduate, 29% graduate, 27% postdoctoral fellows, 36% faculty, and 7% other. The majority (65%) listed cognitive neuroscience as their area of training, followed by 33% listing psychology, 32% network neuroscience/connectomics, 16% clinical, 12% engineering, and 10% mathematics/statistics. On average, respondents reported having worked in the field of brain imaging for 9 years, although the spread of the responses varied widely, from 5% saying that they had only been working for about 1 year, up to 12% saying they’ve been working in the area for over 20 years. In terms of software used, most respondents reported using FSL (50%), followed by SPM (45%), AFNI (18%), and other (27%), with responses being nonexclusive. Most respondents reported using ICA in their analysis sometimes (35%), very often (16%), or always (2%), although a sizable group report using it only rarely (29%) or never (18%). Likewise, most respondents reported using brain parcellations in their work very often (40%) or sometimes (33%), with a small group reporting using it always (11%), relative to rarely (10%) or never (6%). Most respondents reported conducting research using both task fMRI and resting-state functional connectivity (43%), followed by resting-state functional connectivity–based only (36%), and task-based only (22%). Note that we focus here on large-scale networks defined using resting-state fMRI, as this is the most widely used experimental paradigm for mapping and understanding large-scale networks of the human brain.

Participants’ responses were manually coded, and the language was unified (e.g., “somatic” and “somato” were considered the same response) by two independent coders. Data were initially explored in terms of percent agreement. Here, we limit our discussion to those images for which there was the most and the least amount of agreement between responses.

**Figure 1.**
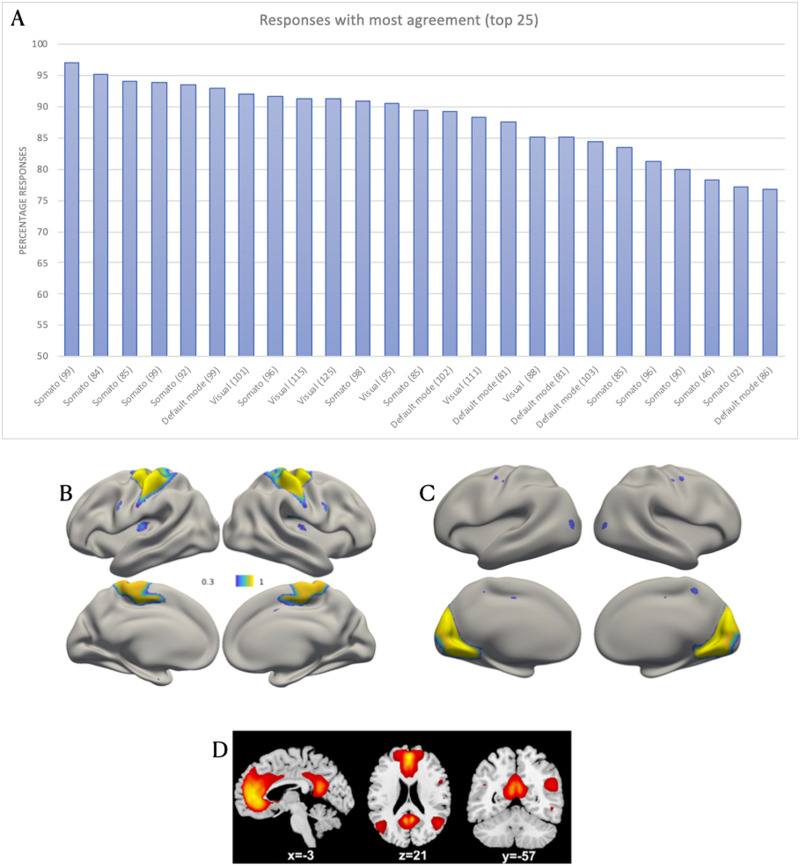
Survey responses with the most agreement across raters. (A) Percentage responses of the top 25 responses in terms of percentage agreement. The vertical number in parentheses next to the response term corresponds to the total number of respondents for that particular image. Each network image is associated with a single bar. Sample images with the largest amount of agreement for (B) the “somato network” (96.97%, *n* = 99), (C) the “visual network” (92.08%, *n* = 101), and (D) the “default network” (92.93%, *n* = 99).

Three networks were identified by consensus in the upper quartile of images (Figure 1). The largest amount of agreement occurred for images that were identified as “somato network.” Of the 25 images with the largest amount of percentage agreement across participants, 13 were identified as “somato network.” The remaining 12 were evenly split between “default network” and “visual network.” Of these three networks, a functional or cognitive nomenclature was provided, as opposed to a neuroanatomical network name (e.g., “occipital”).

**Figure 2.**
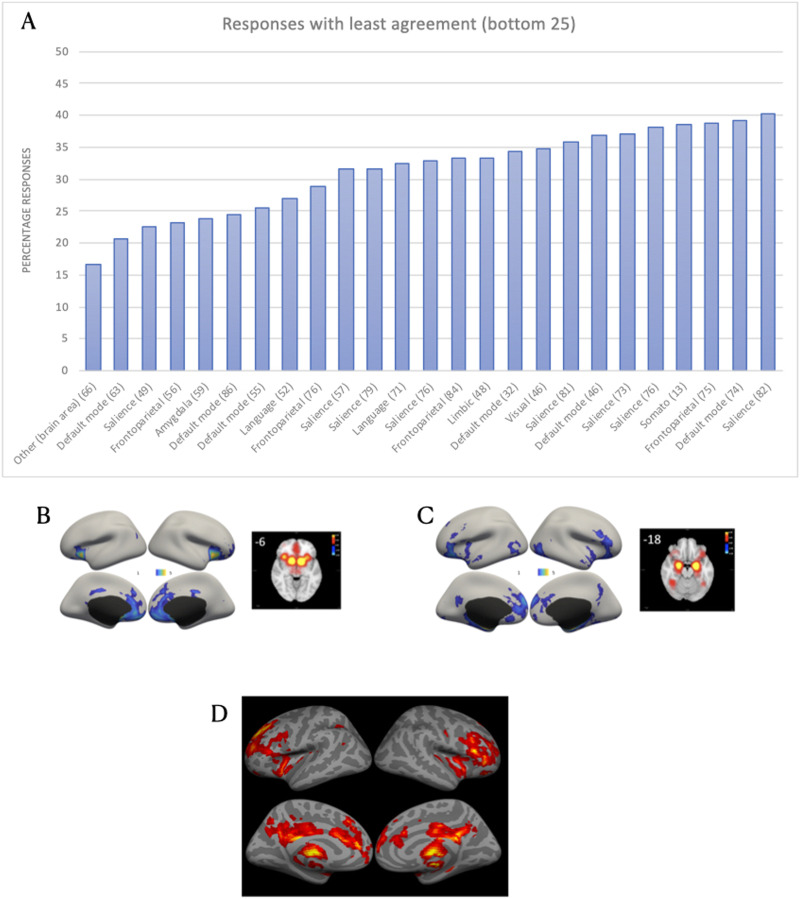
Survey responses with the least agreement across raters. (A) Percentage responses of the bottom 25 responses in terms of percentage agreement. The vertical number in parentheses next to the response term corresponds to the total number of respondents for that particular image. Each network image is associated with a single bar. Sample images with the least amount of agreement. (B) Sample image 13 received a label of “other” (16.67%, *n* = 66). (C) Sample image 12 received a label of “default mode network” (20.63%, *n* = 63). (D) Sample image 88 received a label of “salience network” (22.45%, *n* = 49).

By contrast, when we look at the 25 images with lowest percent agreement in responses, the pattern of results is rather different (Figure 2). All of the lures were included in the images that received 50% or less response agreement. Unlike network labels with high agreement, there was a greater heterogeneity of terms used in the lower quartile of responses. Specifically, 8 of the images were labeled as “salience network”, 6 as “default network,” 4 as “fronto-parietal network,” 2 as “language network,” 1 as “visual network,” 1 as “limbic network,” 1 as “amygdala network,” 1 as “somato network,” and 1 as “other.” In this subset of responses, there was a mix of cognitive/functional terms (such as ‘salience’ and ‘default’) and neuroanatomical terms (such as ‘fronto-parietal’ and ‘amygdala’). Finally, it is worth noting that the number of responses in this lower quartile was rather low (*M*_responses_ = 63.0, *SD* = 17.80), compared with the top 25 (*M*_responses_ = 93.36, *SD* = 14.55). It is likely that the comparatively lower number of responses reflects participants’ hesitance or uncertainty as to whether the relevant image constituted a (canonical) network or, if it did, how it should be labeled.

Cautiously, the results of this initial survey could be interpreted as revealing a preference among scientists to name large-scale brain networks according to their putative cognitive functions. They also suggest a certain degree of agreement regarding at least three canonical networks. These networks include two that are spatially contiguous within the somatomotor and occipital cortices. The third network, the default network, was the only spatially distributed network identified reliably. Of note, these three networks appear in the initial anatomically based taxonomy proposal, which delineated “occipital,” “pericentral,” and “medial fronto-parietal” networks (Uddin et al., 2019).

There was much lower consensus on network labels for all other spatially distributed large-scale brain networks, including the “salience” and “fronto-parietal” networks. While it is difficult to run inferential statistics on these qualitative data, there is much more that could be explored from these results. We have made the survey data publicly available within the Open Science Framework (Spreng, 2022; https://doi.org/10.17605/OSF.IO/3FZTA). Nevertheless, this initial exploration suffices to support the claim that while there is some degree of agreement among scientists regarding the labeling and identification of a few large-scale brain networks, there is a substantial amount of disagreement, raising concerns about the consistency with which results are interpreted, as well as concerns regarding reproducibility in the field more generally. A lack of agreement about basic network definitions will complicate any attempt at replication. These problems could be alleviated by a more standardized nomenclature. In what follows, we will detail considerations for moving beyond these arbitrary naming conventions, and for developing a universal taxonomy that can help to move the field toward greater clarity and consensus in characterizing ensembles of brain regions with more reliable concision.

## CHALLENGES TO AND CONSIDERATIONS FOR BUILDING A UNIVERSAL TAXONOMY OF LARGE-SCALE BRAIN NETWORKS

The results of the survey suggest that there is a potential for consensus around a taxonomy of large-scale brain networks. However, many challenges remain in building further consensus around nomenclature, particularly for spatially distributed networks interposed between the heteromodal and distributed default network on the one hand, and unimodal, spatially contiguous, somatomotor and occipital networks on the other. WHATNET identified five interrelated issues that require careful consideration in building a universal taxonomy of large-scale brain networks. First, the spatial scale and resolution of any network must be considered, as well as how networks are organized hierarchically. This entails a complete description of how a network is defined (e.g., using full correlation, partial correlation, or other approaches). Second, there is substantial variability in network topography between individuals. This variance is observable across the typical healthy young adult populations included in many studies, varies systematically in the context of life-span development, and may become more complex to characterize in clinical populations. Third, brain dynamics, nonstationarity, and contextual effects (such as task-related reconfiguration) have a profound influence on observable network ensembles. Fourth, large-scale brain networks have been reliably demarcated spatially in cortex, but these topographies are incomplete without corresponding subcortical structures that are often ignored in widely used parcellation schemes. Fifth, the investigation of large-scale brain networks is heavily biased toward fMRI research methods. While multimodal information provides some support for fMRI observations, a universal network taxonomy would benefit from deeper enrichment of multimodal information. These five issues, discussed in turn, reflect ongoing research initiatives that necessarily inform the development of any network taxonomy.

### Spatial Scale, Resolution, and Hierarchies

A network is defined by its nodes and edges (Bullmore & Sporns, 2009). Nodes should ideally represent the fundamental functional units of the system, and edges represent the interactions between nodes (Rubinov & Sporns, 2010). The fundamental functional units of the brain have been debated for over a century, with individual cells, cortical columns, Hebbian-like cell ensembles, and cytoarchitectonic regions having been proposed (Mountcastle, 1997; Nieuwenhuys, 2013; Varela et al., 2001; Yuste, 2015). Thus, nodes could, in principle, be defined across spatial scales varying over at least five orders in magnitude, from the level of individual cells (∼10^−5^ mm), through populations of functionally related neurons (∼10^−4^ m to 10^−2^ m), to macroscopic brain areas and distributed functional systems (10^−2^ m to 10^−1^ m) (Betzel & Bassett, 2017; Churchland & Sejnowski, 1988). In the absence of a gold standard method for defining the nodes of a large-scale brain network, and in light of the limited spatial resolution of fMRI, many investigators rely on heuristic approaches or data-driven methods, as outlined below.

Network edges can similarly be used to represent structural and/or functional interactions between nodes over a similar range of spatial scales. Structural edges can represent synapses, axons, bundles of axons, or white matter pathways. Functional edges can represent measures of coupled spike trains or calcium signal fluctuations between individual neurons, covarying local field potentials, or statistical dependencies of physiological recordings taken from extended cell populations (Faber et al., 2019). Graph theory offers a simple and powerful framework for modeling the nodes and edges of a network (Bullmore & Sporns, 2009; Fornito et al., 2016; Rubinov & Sporns, 2010), but various network properties can also be understood using other techniques, such as sICA (Beckmann et al., 2005; Calhoun et al., 2001).

The spatial and temporal resolution at which a given neural system is mapped will necessarily constrain the kinds of networks that can be observed. For instance, it is presently not possible to measure entire nervous systems of mammals at cellular resolution, so any such networks can only be mapped within confined patches of neural tissue, precluding an opportunity to study systems with a more widespread anatomical distribution. In contrast, noninvasive methods such as MRI offer a powerful tool for assaying entire brain volumes at macroscopic spatial resolutions on the order of millimeters and the temporal resolution of fMRI on the order of seconds or subseconds. At the spatial and temporal scales currently accessible with MRI, the large-scale brain networks observed are likely to represent long-term attractors of more rapid cellular dynamics that are shaped by underlying anatomical connectivity and prior interregional coactivation histories (Honey et al., 2010; Messé et al., 2014; Smith et al., 2009).

A particular challenge for macroscale neuroimaging is that there currently exists no gold standard for defining network nodes. This is fundamentally a question of brain parcellation. Under ideal conditions, a network node should correspond to a functional brain area, which may be defined as a contiguous patch of neural tissue that shares homogenous functional specificity, connectivity, architectonics, and topography (Felleman & Van Essen, 1991; Van Essen & Glasser, 2018). Since Brodmann (Brodmann, 1909) first parcellated the brain into distinct cytoarchitectonic regions, numerous investigators have attempted to identify the boundaries between functionally specialized areas and nuclei, both in cortical and noncortical regions (Gordon et al., 2016; Yeo et al., 2011), often leading to conflicting parcellation schemes. Indeed, some have questioned the very existence of discrete brain areas (Lashley & Clark, 1946; Salehi et al., 2020a), and many cellular, molecular, and functional properties of the brain appear to follow spatially continuous gradients (Huntenburg et al., 2018; Shafiei et al., 2020), although statistical evidence for reproducible, discrete transitions across neocortical areal boundaries in multiple independent modalities have been observed (Glasser et al., 2016). In the MRI literature, the lack of cellular or molecular probes has led different investigators to deploy various methods and heuristics for defining network nodes, including random parcellations (Craddock et al., 2012), data-driven clustering based on patterns of structural or functional connectivity (Bellec et al., 2010), gradient-detection algorithms (Wig et al., 2014), the use of activation foci from task fMRI (S. Wang et al., 2020) or the co-localization of gradient-defined boundaries in neuroimaging measures of architecture, function, connectivity, and topography (Glasser et al., 2016). These methods vary in the degree to which they capture the essential properties that define a given brain area, which ultimately determines the composition of an extended functional network. Indeed, the specific way in which nodes are defined will influence the networks that can be identified. For instance, the commonly used automated anatomical labeling (AAL; Rolls et al., 2020) and Desikan-Killiany gyral and sulcal atlases (Desikan et al., 2006) treat the superior frontal gyrus as a single region, yet this gyrus contains several cytoarchitectonically and functionally distinct subregions that cannot be demarcated without a more fine-grained parcellation (Petrides et al., 2012).

The method used to define network edges will also influence the composition of any networks observed with further analysis. In structural networks, edges are typically derived from dMRI tractography, a technique that produces streamlines representing axon bundles. These bundles form the backbone that supports communication between brain areas both at rest and during task performance. While the brain transitions across functional states at a subsecond rate, this structural backbone is not altered at that time scale. Thus, although some relationships are expected between structural and functional networks (e.g., a disruption in the former resulting in a disruption in the latter), these two approaches measure different features of the brain. Furthermore, various analytic choices have an impact on the edges of structural networks as measured by dMRI tractography (Bastiani et al., 2012; Baum et al., 2018; Drakesmith et al., 2015; Jung et al., 2021; Maier-Hein et al., 2017; Oldham et al., 2020; Parker et al., 2014; Sinke et al., 2018; Zhong et al., 2015) (see section Network Affiliations of Subcortical Structures), and the effect of these analytic choices on the accuracy of dMRI with respect to the ground truth obtained from postmortem microscopy is an area of active investigation (Donahue et al., 2016; Hayashi et al., 2021; for review see Yendiki et al., 2022).

In functional networks, connectivity can be defined using statistical techniques (e.g., correlation or coherence) for quantifying a dependence between bivariate or multivariate time series (Friston, 1994). The choice of a specific coupling measure has a significant impact on network structure. The product-moment correlation coefficient is the most widely used in the fMRI literature. Correlation-based networks are sensitive to indirect, polysynaptic connections and tend to be strongly clustered (Zalesky et al., 2012). The use of partial correlations can remove these indirect effects but, when applied to large networks, can be too aggressive and remove important network structure (Reid et al., 2019; Zalesky et al., 2012). On the other hand, the presence of colliders (or common effects) in a network can result in false positives with partial correlation (Sanchez-Romero & Cole, 2021). An additional complication arises from the fact that just because two nodes are each highly correlated with another node does not necessarily imply that those two nodes are highly correlated with each other (Erhardt et al., 2011). These nuances and variations in methods for node and edge definition are compounded by the myriad ways available for processing and denoising fMRI data (Ciric et al., 2017; Parkes et al., 2018), which can yield divergent estimates of network connectivity.

The analysis of network organization is typically based on the idea that a network can be understood in terms of a set of separable “communities” (or “modules”). Note that although the terms ‘communities’ and ‘modules’ are sometimes used interchangeably, they need not be the same (Stanley et al., 2019). A given node belongs to one and only one community in most formulations. Whereas this assumption is a reasonable starting point, there is no inherent reason that biological networks should be organized in this precise manner (Palla et al., 2005). As analogy, a specific gene may participate in many metabolic pathways and thus be better understood as belonging to more than one community. Likewise, hub regions in the brain are thought to dynamically affiliate with disparate clusters of brain regions in a context-dependent fashion (Cole et al., 2013; Pessoa, 2014).

In the past decade, several research groups have investigated the overlapping nature of brain communities (Faskowitz et al., 2020; Jo et al., 2021; Najafi et al., 2016; Yeo et al., 2014). Among other findings, this work has revealed that select brain regions connect both within their community, and also across communities. In this way, communities have somewhat fuzzy boundaries, but it is also clear that specific nodes can play important roles in multiple communities. More broadly, this research encourages discussions that evaluate common assumptions in understanding large-scale brain networks. Are hard partitions the appropriate mathematical language, or would it be valuable to adopt a notion of “gradients of affiliation” that embraces a more continuous, albeit complex, characterization of network architecture? Indeed, even within a discrete brain region there is evidence of functional multiplicity, such that distinct yet overlapping gradients of functional organization can be identified (Haak & Beckmann, 2020). We return to these issues in the final section where we provide recommendations for dealing with these ambiguities.

For now, these issues are illustrated by considering one popular approach for functional parcellation of fMRI data: sICA. sICA is a data-driven decomposition method that can be applied to fMRI data to decompose the data into a collection of spatial signal sources, for example, fMRI signals from brain networks and from motion effects and artifacts, that mix together to generate the measured fMRI data. There are many applications of sICA for fMRI, including data denoising, data reduction, and investigating functional connectivity (Abou-Elseoud et al., 2010; Beckmann et al., 2005; Calhoun et al., 2001; Chong et al., 2017; Hu et al., 2020; Kiviniemi et al., 2009; Salimi-Khorshidi et al., 2014). Here we discuss how sICA has typically been applied in the service of large-scale brain network identification.

For functional connectivity analysis, sICA is typically applied to group fMRI data created by temporally concatenating the fMRI data across subjects. Resulting group sICA spatial maps then represent a data-driven soft functional parcellation, with spatial maps representing spatially independent sources of the data (neural and nuisance signals) that are common to all participants. To investigate intersubject variability in between- or within-network connectivity, this parcellation is projected back into each participants’ fMRI data to compute subject-specific spatial maps and time courses for functional connectivity analyses. In this two-step procedure, the group sICA can be used to define discrete neural elements and can be tuned to different spatial scales observable with fMRI, from high-dimensional parcellations of the brain into individual or bilateral localized brain regions (∼mm to few cm) to modules or subnetworks comprised of a few nodes (few cm to ∼10 mm), to widely spatially distributed large-scale networks (whole brain ∼10 cm). The projection step, for example, using dual regression (Nickerson et al., 2017), extracts temporal courses for each map in the parcellation scheme from each subjects’ data, which may then be used to compute subject-specific spatial maps that approximate the unique configuration of the networks represented in the group sICA in each participant. The subject-specific temporal courses can be used for network modeling, for example, to assess between-network connectivity, while the spatial maps capture intersubject variability in connectivity of individual networks that can be assessed to compute group differences or relationships with nonimaging variables. For network modeling using the network time courses, the same issues raised above related to edge selection apply (e.g., full correlation vs. partial correlation; Pervaiz et al., 2020; Smith et al., 2009).

The spatial scale of the group sICA parcellation is determined by the group sICA model order parameter, or the number of components estimated by sICA. For group sICA of fMRI data, low model orders of ∼20 result in a parcellation into large-scale brain networks (Beckmann et al., 2005; Kiviniemi et al., 2003; Smith et al., 2009), whereas higher model orders of ∼30–70 parcellate the brain into subnetworks, and the highest model orders (100–300+) implement a fine parcellation into individual unilateral and bilateral brain regions as well as subnetworks that do remain stable across a wide range of higher model orders (although at extremely high model orders may eventually fractionate; Smith et al., 2009, 2013). As an aside, care must be taken to balance the desired spatial scale against the particular subnetworks that may be of interest. Most sICA methods applied to group-level fMRI data implement a principal component analysis (PCA) for data reduction prior to the sICA. This step identifies orthogonal signals of interest that explain the most variance in the fMRI data, starting with the signal accounting for the most variance and progressing down to signals that account for small variance. The data are reduced by discarding the components that exceed the model order of the sICA. As such, it frequently happens that brain networks that do not account for enough variance to make it through the PCA reduction step are discarded and do not show up in lower model order sICA spatial maps. As the model order increases, we then see not only fractionation of brain networks into subnetworks/regions, but also the appearance of previously unobserved networks that were not observed at lower model orders (Abou-Elseoud et al., 2010; Hu et al., 2020). This is particularly true for subcortical and brainstem networks. For example, in Abou-Elseoud et al. (2010), the basal ganglia network does not appear until model order 40 (their Figure 2). Techniques that do not apply PCA, such as independent vector analysis (Lee et al., 2008), and those that can account for low variance networks, such as snowball ICA (Hu et al., 2020) and recursive ICA (Iraji et al., 2022), obviate this issue.

**Figure 3.**
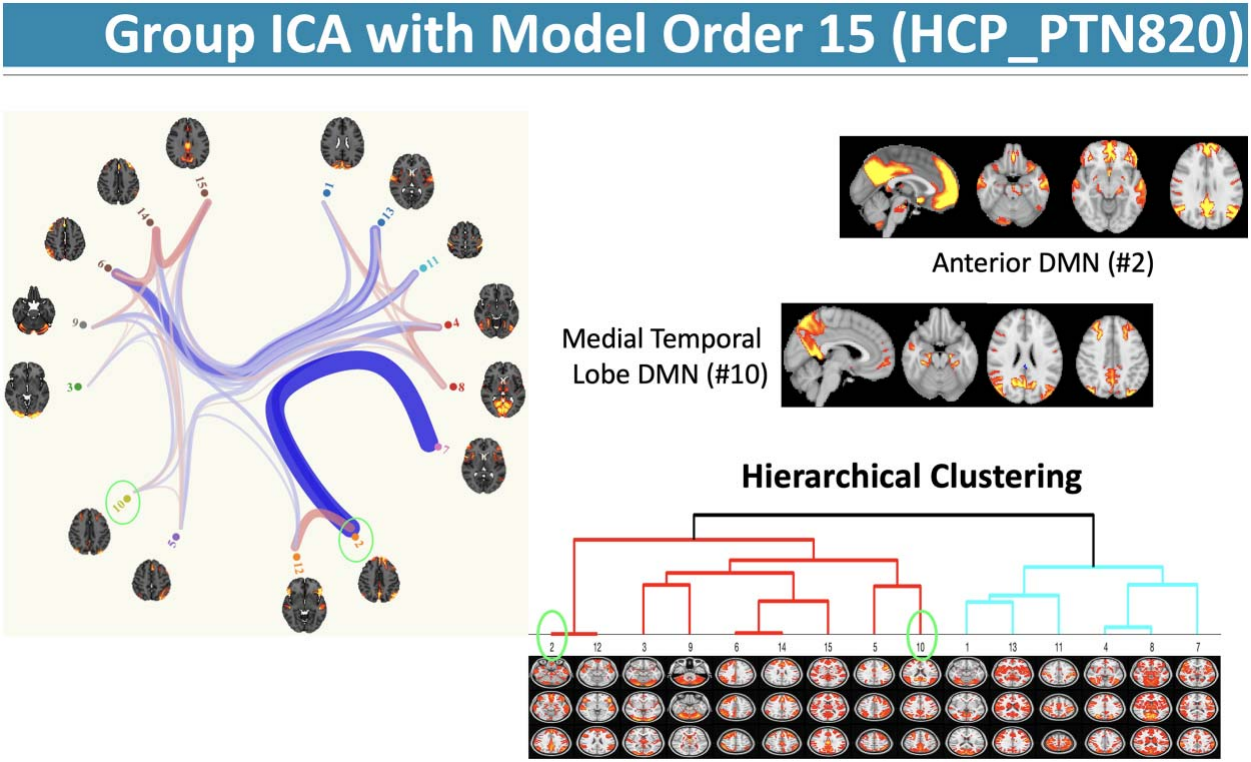
Default network hierarchies. Two subsystems of the default network (default ‘mode’ network, DMN) are identified in the group sICA with model order = 15: an anterior (dorsomedial prefrontal) subsystem and a medial temporal lobe subsystem. Hierarchical clustering shows they cluster together at the second level of the tree. Although these two systems are related, they are also strongly connected with other networks, for example, anterior default network is linked with the inferior frontal-opercular system (#12; also referred to as the salience network); medial temporal lobe default subsystem is linked with a left fronto-parietal control network (#5; also referred to as the central executive network). Although both components are likely part of an extended default network, there is no clarity for a “superordinate” system that best exemplifies the default network. Component 2 may be recognized as the more canonical network as the focus of subsequent analyses, with Component 10 discarded. This example highlights the need for a network taxonomy that addresses subnetworks at multiple spatial scales.

To demonstrate the links between parcellation across spatial scales and model orders, we consider the open access sICA-based group-level functional parcellations (Smith et al., 2013) distributed by the Human Connectome Project (Van Essen et al., 2013). Figure 3 shows that in the parcellation from model order = 15 (from HCP_PTN820), there are two networks that comprise regions consistent with the default network. The question then is: Which one best characterizes the network, or do both represent fractionated subnetworks of the default network? Prior work has identified subnetworks of the default network from resting-state fMRI (dorsomedial prefrontal subsystem/anterior default network; medial temporal lobe subsystem/ posterior default network) with converging evidence of dissociable cognitive processes from task fMRI (Andrews-Hanna et al., 2014). However, in the absence of a universal taxonomy, default subnetwork labels may be interpreted differently across investigators. Model order in sICA may further complicate the matter, with higher order models further fractionating subsystems, potentially leading to idiosyncratic labels and challenges with integrating new discoveries into the existing scientific corpus.

**Figure 4.**
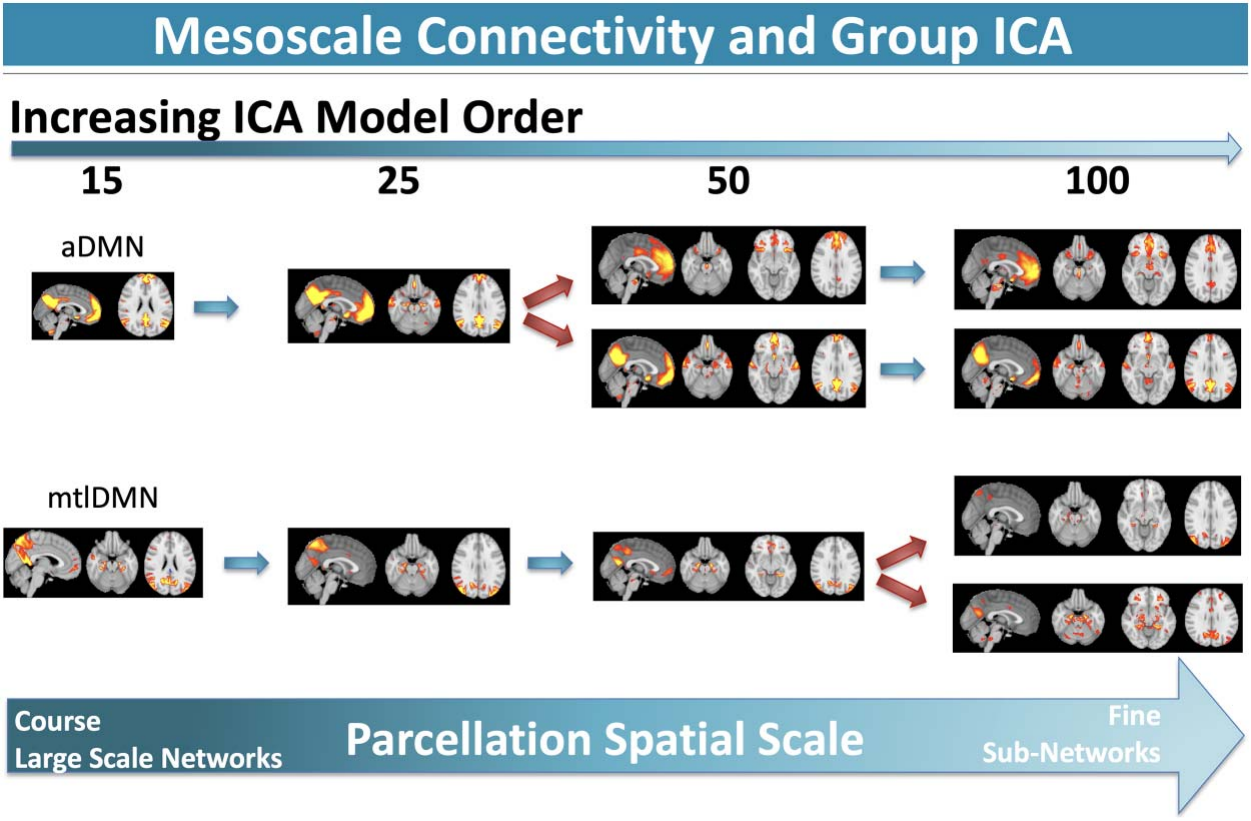
Default network fractionation. At model order = 15, the default network (DMN) is fractionated into two subnetworks: anterior and medial temporal lobe. These two subnetworks are stable across model order = 25, but the anterior default network (aDMN) fractionates into two further subdivisions, a dorsomedial prefrontal component and a ventromedial prefrontal component, which are stable at model order = 100. The medial temporal lobe default network (mtlDMN) fractionates at model order = 100 into two components, which is best characterized by a neuroanatomical dissociation in medial parietal cortex, with a precuneus mtlDMN subnetwork and a posterior cingulate cortex mtlDMN subnetwork. Further fractionation is observable at higher model orders (200–300, not shown). Depending on model order, several components could plausibly comprise the default network. The reliable application of labels to these subnetworks is inconsistent throughout the literature with no guiding taxonomy.

To illustrate these issues, we again used the ICA parcellations provided by the Human Connectome Project. We selected the anterior default network as the “root component” to track through the other parcellations across model orders. To track across parcellations, we concatenated the subject-level time courses for the root component together with the subject-level time courses from each of the other model orders, 25, 50, and 100. We then used FSLNets to implement hierarchical clustering using Ward’s minimum variance method on each of the full correlation matrices. The resulting clusters and dendrograms were examined for components that clustered closely with the root component for each model order and for the appearance of default network subnetworks at finer scale parcellations, where subnetworks were identified as those that had spatial overlap with parts of the root component. Figure 4 shows how these two networks persist and/or fractionate as the group sICA model order increases, from the coarsest parcellation at lowest model order to the finest parcellation at high model order. Both anterior and medial temporal lobe default subnetworks are stable as the model order increases to 25. However, at model order 50, the anterior default network further fractionates into dorsal and ventral prefrontal subsystems (Hiser & Koenigs, 2018), while the medial temporal lobe subsystem is represented as a single default subnetwork. At model order 100, the medial temporal lobe subsystem is split into two additional components, while the dorsal and ventral prefrontal default components of the anterior default network subsystem remain stable. These systems remain stable and/or are further split at the highest model orders (200, 300; not shown). This multiscale organization may reveal important information about hierarchical brain organization. However, this example also shows how method variance (i.e., model order) and experimenter choice interact to impact mesoscale functional connectivity results, presenting real challenges for node definition, parcellation, network and subnetwork identification, and conceptual clarity for network labels and nomenclature.

### Interindividual Variability

**Figure 5.**
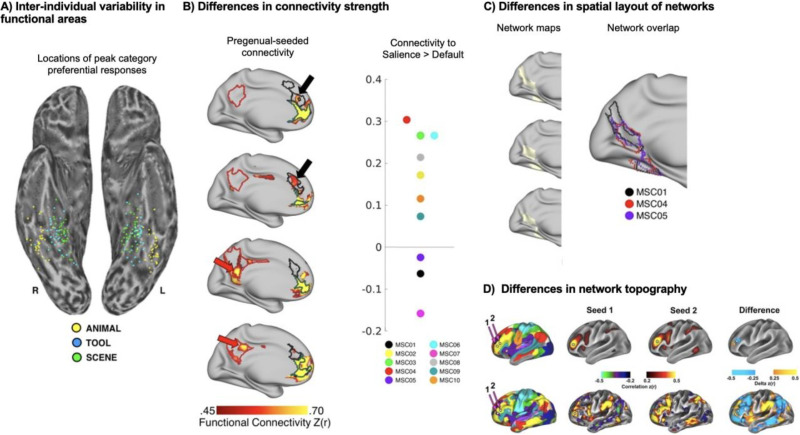
Forms of interindividual variation in functional neuroanatomy and large-scale brain network topography. (A) Task-responsive cortical areas, which comprise large-scale brain networks, vary in their spatial location across individuals. (B) Similar network components are present across individuals, but differ in magnitude of associations. (C) Large-scale brain networks differ between people in size and position. (D) Whole-brain functional connectivity, resulting from small differences in seed placement, can reveal dramatically different network topographies between people (Laumann et al., 2015). Adapted figure panels A from Stevens et al. (2015), B–C from Gordon and Nelson (2021), with permission.

Functional neuroanatomy varies spatially across individuals over and above preprocessing procedures that normalize neuroimages to a standard space. This has been recognized for over 20 years by cognitive neuroscientists who engage in task-based functional localization to characterize regional brain function (e.g., Kanwisher et al., 1997, fusiform face area). For example, the parahippocampal “place” area can vary by up to 20 mm along a rostral-caudal axis between individuals (Figure 5A; Stevens et al., 2015). Just as brain regions vary in location across individuals, so does large-scale brain network topography (Kong et al., 2019). A discussion of large-scale brain network taxonomy requires an understanding of variation in these systems across individuals. It also requires a clear assessment of the degree to which this variation impacts our ability to identify common networks across individuals (Dworetsky et al., 2021).

Individual variability interacts with questions of taxonomy in at least two ways. First, networks are often first identified based on evidence that a particular set of regions are linked to one another consistently across individuals. For example, if, in every person we examine, we find links between the posterior cingulate, medial prefrontal cortex, and angular gyrus, we increase our confidence that this is a ‘network’ entity and apply a label to it (e.g., “default network”). Recent work on individual variability shows that the boundaries between the default network and other networks can be readily identified within individual participants (Braga et al., 2019; Braga & Buckner, 2017; DiNicola et al., 2020; Gordon et al., 2020).

Second, in the face of interindividual variability, we need an approach for how to determine correspondence across individuals in order to implement taxonomies in practice. fMRI functional connectivity studies suggest that while some regions of the brain appear to be largely similar in their network topography across individuals, others show pronounced individual differences (Kong et al., 2019; Laumann et al., 2015; Mueller et al., 2013; Seitzman et al., 2019). These studies have found that association regions, especially in lateral prefrontal cortex and near the temporo-parietal-occipital junction, tend to exhibit the most variable spatial topography of the cortex, with much lower variation seen in sensory and motor regions. Given this variation, how can we determine that a network is the same entity across different individuals or groups? If we find an individual missing specific components of Network A or with a spatial topography similar but slightly displaced relative to Network A, would it be accurate to call this Network A? In what ways, and to what extent, can a network vary but still represent the same underlying entity? To what extent should we expect certain networks to only be expressed in some individuals but not others?

These questions are not purely theoretical. For instance, following complete hemispherectomy—a surgical procedure wherein an entire cerebral hemisphere is removed—the lone remaining hemisphere can exhibit network properties typically observed in individuals with fully intact brain structures (Ivanova et al., 2017; Kliemann et al., 2019). Individuals with extensive cortical loss due to prenatal stroke can exhibit intact behavioral function, accompanied by networks that are entirely preserved but displaced away from the stroke (Laumann et al., 2021). Even well-studied networks that appear in roughly similar locations in almost every person do not correspond exactly anatomically (e.g., the default network, Braga & Buckner, 2017; or somatomotor networks, Gordon et al., 2017c). A recent quantitative examination of intersubject variability of 14 large-scale brain networks finds that while networks exhibit a common core, consistency across individuals falls off sharply, especially for networks that include prefrontal and parietal cortex (Dworetsky et al., 2021). Due to individual variation in functional neuroanatomy, uniform application of standard parcellation schemes will result in lower sensitivity and specificity (Fedorenko, 2021; Gilmore et al., 2021; Salvo et al., 2021; Smith et al., 2021).

Implementation of a robust network taxonomy requires a way to estimate and compare networks across individuals while respecting interindividual variability. Below, we discuss key factors when considering the relationship between universal large-scale brain networks and interindividual variability: (1) methods that capture individual brain networks, (2) validation of these measures and sources of potential errors, (3) what studies of individual brain networks reveal about common forms of individual variability, and (4) how brain networks differ across different subsets of the population (based on age or disease status).

Many approaches have been developed to estimate individual variability in brain network organization. One class of approaches applies data-driven techniques (e.g., clustering) to a large quantity of data at the individual level (Braga & Buckner, 2017; Gordon et al., 2017c; Laumann et al., 2015). However, this so-called ‘precision functional mapping’ approach requires a large quantity of data from each participant, although less data may be needed with multiecho fMRI data acquisition (Lynch et al., 2020a). In addition, each participant is analyzed separately, so network correspondence between participants is not enforced, and post hoc network assignment can be uncertain in some circumstances.

Group ICA (Du & Fan, 2013; Mejia et al., 2020) and independent vector analysis are two data-driven approaches that converge with group-level network results while capturing individual-specific variance in subspaces (Lee et al., 2008; Michael et al., 2014; Vij et al., 2018). Group ICA with back-reconstruction (Erhardt et al., 2011) or dual regression (Nickerson et al., 2017) are popular approaches that project group ICA spatial maps into, or apply multivariate spatial regression of group ICA spatial maps against, individual-level data, respectively, to identify individual-specific networks corresponding to the group ICA networks. More recently, a second class of approaches estimates individual-specific networks by constraining them to a spatially similar group-level prior (Chong et al., 2017; Dworetsky et al., 2021; Gordon et al., 2017a; Harrison et al., 2015; Kong et al., 2019; D. Wang et al., 2015). This class of approaches allows the reliable estimation of individual-specific networks and preserves network correspondence across participants. However, the use of a group-level prior might restrict the flexibility of networks to vary across participants. Approaches can vary along the full spectrum, from completely data-driven to strongly prior-driven. Priors can be delimited in a variety ways, such as constraining the size, shape, number of nodes, topography or location of a network, which will have different implications for the resulting network characterization. Even when using a group-level prior, some approaches can accommodate large deviations across participants. Finally, a third class of approaches estimate intersubject variability continuously at the voxel or vertex level (Mueller et al., 2013; Seitzman et al., 2019), making no assumption about the number or form of underlying networks. However, this approach assumes voxelwise/vertexwise correspondence across participants, which is a strong assumption given pronounced individual differences across participants. All three of these classes of approaches exhibit some weaknesses, and none of the extant techniques fully match brain areas across individuals. In cases with relatively little data across participants, an appropriate solution is to employ techniques that match each individual to a detailed, areal-level group reference atlas (e.g., Kong et al., 2021; Chong et al., 2017), with the caveat that such approaches will never identify individual-specific areas that are absent from the reference atlas (cf. Dworetsky et al., 2023; Seitzman et al., 2019). We anticipate that future work will improve current techniques with the aim of minimizing these identified weaknesses.

What is the evidence that the interindividual network variability is reliable? First, these networks are highly replicable across sessions within the same participant (Kong et al., 2019; Laumann et al., 2015; D. Wang et al., 2015). Second, task-evoked fMRI activations align well with the idiosyncratic network topography in individual participants (Braga et al., 2020; Chong et al., 2017; DiNicola et al., 2020; Gordon et al., 2017a; Seitzman et al., 2019). Third, individual-specific network topography is heritable (Anderson et al., 2021). Finally, individual-specific network topography can better predict behavioral traits of individual participants (Bijsterbosch et al., 2019; Feilong et al., 2021; Kong et al., 2019; Sassenberg et al., 2023; Seitzman et al., 2019; Feilong et al., 2021).

However, care is needed in the measurement of individual variability. In addition to reflecting variation in large-scale brain networks, variability in functional connectivity can also be induced by nonneural sources such as motion (Power et al., 2012, 2014, 2020; Satterthwaite et al., 2012; Van Dijk et al., 2012), respiration (Birn et al., 2008; Chang & Glover, 2009), sampling variability (Laumann et al., 2015, 2017), and signal loss due to acquisition parameters, head shape or head position. For example, while functional networks have been described as shifting from a more local to a more distributed pattern from childhood to young adulthood, some of this variation is likely to be caused by head motion, which induces distance-dependent artifacts (Nielsen et al., 2019; Power et al., 2012; Satterthwaite et al., 2012). Respiration has been associated with global BOLD signal changes, which introduces one of many sources of artifact into fMRI functional connectivity estimates (Lynch et al., 2020b; Power et al., 2017, 2020). Additionally, the fMRI BOLD signal is quite noisy and autocorrelated; a fair amount of data is needed to counteract this sampling variability and reach high reliability at an individual subject level (Elliott et al., 2019; Laumann et al., 2015, 2017; Noble et al., 2017). These factors differ across brain regions concurrent with properties of the underlying BOLD signal and MRI measurement method. Many popular fMRI sequences result in substantial signal loss and distortion near tissue boundaries and reduced signal further from the receiving coil, leading to difficulty in accurately measuring functional networks in certain brain regions, particularly impacting orbitofrontal cortex, the ventral temporal lobes, and subcortex (Noble et al., 2017). Caution is warranted in interpreting variation in functional networks if these nonneural sources of variation are not adequately addressed. A growing number of papers have assessed the ability of different acquisition, preprocessing, and denoising paradigms to address these artifacts (Burgess et al., 2016; Ciric et al., 2017; Glasser et al., 2018; Kundu et al., 2017; Lynch et al., 2020a; Power et al., 2018, 2020; Salimi-Khorshidi et al., 2014).

When such confounds are minimized, it becomes evident that several different forms of interindividual variability are present in brain networks (Figure 5). The most commonly studied form of interindividual variation is variation in the magnitude of connectivity between brain regions of a network (Figure 5B). Individual differences in connectivity strength are often taken as an outcome metric of interest, to be associated with external states or traits (Marek et al., 2020; Stevens & Spreng, 2014). However, when connectivity strength is used to define networks, as discussed here, large interindividual variabilities in connectivity strength between networks can introduce ambiguity about network membership (Bijsterbosch et al., 2020; Gordon & Nelson, 2021).

A second form of interindividual variation is variation in the spatial position and extent of network nodes (Figure 5C) (Bijsterbosch et al., 2019; Braga et al., 2020; Braga & Buckner, 2017; Chong et al., 2017; Dworetsky et al., 2023; Gordon et al., 2017a, 2017b, 2017c; Greene et al., 2020; Harrison et al., 2015; Kong et al., 2019, 2021; Kraus et al., 2021; Laumann et al., 2015; Marek et al., 2018; Seitzman et al., 2019; D. Wang et al., 2015; Xue et al., 2021). Such variations can take the form of areal expansions, contractions, or displacements that lead to variation in the exact positions of network borders across individuals (Dworetsky et al., 2023; Gordon et al., 2017b). More extreme individual spatial variations can relocate a node outside of the initialized parcel boundary, with no spatial overlap with other sample participants (Chong et al., 2017; Gordon & Nelson, 2021). Overall, spatial variation appears to contribute more to individual differences in brain networks than variations in connectivity strength (Bijsterbosch et al., 2019). This source of variance is not respected with the application of standard parcellation schemes, resulting in poorer network estimation. This can introduce systematic bias when considering groups of individuals that deviate from the population used to create the parcellation scheme.

Finally, nodes can exhibit topographical variation across individuals (Figure 5D). Single cortical areas representing network nodes can be split into multiple discontinuous regions in some individuals, while still clearly exhibiting the same properties of the unified area (Glasser et al., 2016). Individual-specific brain networks also exhibit ectopic intrusions, in which a punctate region within a brain network has strong, idiosyncratic connectivity with a different network (Dworetsky et al., 2023; Gordon & Nelson, 2021; Laumann et al., 2015; Seitzman et al., 2019). All individuals exhibit some form of topographical variation in their large-scale brain networks (Dworetsky et al., 2023). However, it is unclear if these violations of regional spatial contiguity reflect a differential sampling of regions on different hierarchical scales.

Each of these forms of variation is important to consider when building or applying a taxonomy of large-scale brain networks, and emphasizes the value of employing individual-level network definitions. In a given individual, variation in functional connectivity strength may result in a canonical network node to “fall out” of the network or be incorporated into a different network. Spatial variation in networks may create the appearance that a network node is absent or disconnected when it is actually mis-localized by standardized parcellation schemes. Topographical variations may create apparent extra network nodes not typically present in most individuals. Any taxonomy must reflect the central tendency network characteristics of the general population, but also be flexible enough to accommodate connectional, spatial, and topographical variations found across individuals.

A recent approach to incorporate individual-level data into our understanding of group-level networks comes from Dworetsky and colleagues (Dworetsky et al., 2021). The authors created individual-level network maps of 14 common large-scale brain networks for each young adult participant in several large datasets. They then used these individual-level maps to create a probabilistic atlas of brain networks (publicly available at: https://github.com/GrattonLab/Dworetsky_etal_ConsensusNetworks). This information can be used to evaluate the range of network associations that are commonly found for different cortical locations. This information can improve future group and individual analyses by allowing researchers to either restrict their analyses to high consensus locations across individuals, or to identify the range of networks associated with variable network locations.

In addition to the interindividual variability in large-scale brain network topography seen in typical samples of young healthy adults, there is also variability across the human life-span and in clinical populations. Developmental differences are often observed in within- and between-network connectivity. For example, developmental brain maturation entails a gradual change from more diffuse connectivity patterns in young children to more clustered systems in young adulthood (Gu et al., 2015). This pattern appears to reverse in aging, where we observe dedifferentiation of connectivity in older adults, with lower within-network and greater between-network connectivity (Damoiseaux, 2017; Setton et al., 2023; Wig, 2017). Other examples are clinical disorders that have been described as disconnection syndromes, such as Alzheimer’s disease (Delbeuck et al., 2003) and schizophrenia (Bullmore et al., 1997), which both show aberrant default network functional connectivity (Damoiseaux et al., 2012; Whitfield-Gabrieli et al., 2009). An important consideration in this context is whether such age- and/or disease-related connectivity differences fundamentally affect brain network organization. If they do, how should future studies consider potential differences in large-scale brain network organization across the life-span or among clinical groups (see Zhang et al., 2021, for review)?

The current literature shows that most canonical brain networks can be detected across different age groups and clinical populations, even if functional connectivity strength may be attenuated in certain cases (Damoiseaux et al., 2008; Greicius et al., 2004; Uddin et al., 2008a). Moreover, the overall spatial organization of large-scale brain networks appears quite stable across individuals with or without psychiatric disorders, with group differences being relatively subtle (Spronk et al., 2021). Like the observation in typical young adults (Dworetsky et al., 2021), variability in children and older adults can be seen near the boundaries of large-scale brain networks, with the core network regions remaining relatively stable (Cui et al., 2020; Han et al., 2018). Most studies that compare groups examine differences in functional connectivity strength using existing parcellations of predefined brain systems, which are commonly derived from healthy young adult samples (Yeo et al., 2011). To ensure comparison of the same brain systems across groups, researchers may consider restricting functional connectivity evaluations to core network regions, as those proposed by Dworetsky et al. (2021).

Recent work has extended these findings to map networks probabilistically in children and teens from the Adolescent Brain Cognitive Development study (Hermosillo et al., 2022). However, potentially interesting information may be lost when solely focusing on signal from core system nodes, as variability across typical young individuals appears mainly due to differences in spatial topography rather than functional connectivity strength (Bijsterbosch et al., 2018). Alternately, methods to individualize parcellations (including totally data-driven or incorporation of a group prior), discussed above, can also be applied to groups other than young adults. This approach has been successfully applied to examine age differences in the functional architecture of the brain while respecting interindividual variability in large-scale brain network topography (Kantarovich et al., 2022; Setton et al., 2023). Future work should consider, however, whether variance should be benchmarked to normative patterns in healthy young adult brains, or be more flexibly applied to characterize systematic patterns of variance in large-scale brain network functional organization.

### Time-Varying Properties: Dynamics, Nonstationarity, and Contextual Effects on Network Organization

Just as large-scale brain networks can vary in their composition and topography across individuals, they vary within individuals across time as well. Thus, it is important to consider fMRI BOLD as a dynamic signal that varies across multiple levels of temporal resolution. Contextual features of an fMRI scan, such as time of day (Orban et al., 2020), recent experience, and learning can all modulate network properties and their relationship to cognition and affect (Stevens & Spreng, 2014). Additionally, the BOLD signal is dynamic within a single scan. In this next section we discuss how correlation magnitudes fluctuate across time under the assumption that nodes themselves remain relatively stable within an individual, at least over shorter timescales (seconds, minutes), resulting in time-varying dynamics in whole-brain connectome organization. An open question regarding the validity of this assumption remains unresolved.

While time-varying functional connectivity analyses may be susceptible to spurious findings, careful handling of motion and other artifacts, along with implementation of appropriate statistical methods, allows for important insights to be gained using a dynamic approach (Lurie et al., 2020). The question we address here is whether these dynamics should be considered when defining large-scale brain networks. We address changes in network composition both in response to changing cognitive demands (e.g., when performing different cognitive tasks) and on a moment-to-moment level within a particular cognitive context (e.g., during a resting-state scan).

Dynamic functional connectivity approaches are rapidly gaining traction in resting-state fMRI research since the initial observations that over the course of a scan, brain regions can change their connectivity patterns (Chang & Glover, 2010). Several reviews summarize current progress in methodological approaches and describe outstanding issues in this emerging field (Calhoun et al., 2014; Hutchison et al., 2013; Lurie et al., 2020; Reid et al., 2019). Ongoing debates continue regarding the extent to which these dynamics are best characterized as transient phenomena, or reflect an extreme tail of a continuous process (Iraji et al., 2022).

Large-scale brain network organization remains largely stable between rest and task states (Cole et al., 2014; Gratton et al., 2018; Krienen et al., 2014). Data-driven weighted methods (e.g., temporal ICA) treat rest and task activation on a level playing field and find large-scale brain networks during both rest and task states (Glasser et al., 2018). Yet differences are observed, and are thought to be meaningful and systematic (e.g., a result of differing levels of arousal or of the specific cognitive or affective context; Bolt et al., 2017; Cohen, 2018; Gonzalez-Castillo & Bandettini, 2018; Kinnison et al., 2012; McMenamin et al., 2014; Najafi et al., 2017). Much literature focusing on network reconfiguration describes changes in overall network topology, such as the degree of modularity or across-network integration, without probing whether nodes of intrinsic networks change network affiliation across cognitive contexts (e.g., Cocchi et al., 2014; Cohen & D’Esposito, 2016; Spadone et al., 2015; Sun et al., 2020). Other work, however, reports changes in network affiliation that occur when cognitive demands change (e.g., Bassett et al., 2015; Braun et al., 2015; Hearne et al., 2017; Spreng et al., 2013). Recently, it has been reported that network membership of up to 75% of nodes changes across a variety of cognitive tasks. Moreover, the specific cognitive context can be successfully predicted based on patterns of change in network affiliation (Salehi et al., 2020b). Thus, task context is an important feature to consider when characterizing large-scale brain network topography. For example, the default network consists of subnetworks (Andrews-Hanna et al., 2010; Buckner & DiNicola, 2019; Dixon et al., 2017, 2018) that are more distinguishable in terms of community membership during cognitive tasks compared with rest (Dixon et al., 2017; Fornito et al., 2012).

One set of brain regions whose categorization needs particular attention in terms of assignment to intrinsic networks are “flexible hubs” (Cole et al., 2013). These are nodes that connect across several large-scale brain networks (e.g., connector hubs) and whose connections vary as a result of cognitive context. These regions are thought to be critical for integrating across specific task demands in complex cognitive tasks (Bertolero et al., 2015; Cocuzza et al., 2020; Shine et al., 2016). Depending on how network affiliation is defined, these nodes increase their between-network functional connectivity (Gratton et al., 2016) and even change network membership (Bassett et al., 2011) across task contexts. Given these contextual changes in network affiliation, how should these flexible hubs be defined when considering a harmonized taxonomy of brain networks? Probabilistic mapping of network membership in core networks across cognitive contexts is one promising direction for differentiating between network nodes that are stable across cognitive contexts and those that flexibly change their network membership such as flexible hubs; to date this strategy has largely been used to identify consistency across subjects (Dworetsky et al., 2021).

Even *within* a particular cognitive context, the whole-brain pattern of connectivity changes, affecting moment-to-moment cognition (Betzel et al., 2016). How do the relatively short-lived patterns of connectivity aggregate to generate the static connectome organization, and thus the “canonical” large-scale brain networks that we seek to characterize? It is known that the connections with the least amount of time-varying dynamics are those with the strongest correlations in the static connectome (Zalesky et al., 2014). Investigations of connectome states, defined as recurrent, quasi-discrete whole-brain connectivity patterns (derived, e.g., by clustering or hidden Markov modeling), are in line with this observation. Specifically, static organization can be viewed as a “common denominator” that is to some degree present in most functional connectome states, while the individual states express additional state-specific spatial features (e.g., Allen et al., 2014). However, fMRI investigations at a finer temporal resolution further suggest that, at any given moment, a specific combination of the “canonical” large-scale brain networks are coactivated, while the remaining networks are collectively inactive (or deactivated) (Sporns et al., 2021). Thus, the static connectome organization may be primarily driven by short-lived but high-amplitude coactivations (Betzel et al., 2022; Esfahlani et al., 2020). Some interpret these results to suggest that the canonical networks whose strong within-network connectivity dominates the static connectome may be better thought of as recurrent *transient* phenomena, rather than a stable property of the brain (for alternative interpretations, see Ladwig et al., 2022).

In spite of the many varieties of dynamics observed, the evidence suggests that brain networks may reflect minimal “atoms” of connectivity; by and large moment-to-moment cofluctuations respect the membership to canonical large-scale brain networks. In other words, brain regions do not cofluctuate in random sets. Rather, different recombinations of large-scale brain networks describe whole-brain spatial patterns of connectivity from moment to moment, while maintaining the atoms as a relatively continuous feature, thus cumulatively generating the static functional connectome organization.

### Network Affiliations of Subcortical Structures

Variable inclusion of subcortical regions between studies has contributed to differences in network characterization and is a significant factor for consideration in developing a taxonomy. If we define our standardized large-scale brain networks without including subcortical regions, they will not be applicable to studies that include them, and vice versa. Human large-scale brain network identification has largely focused on characterizing networks in the cerebral cortex (Power et al., 2011b; Smith et al., 2009; Yeo et al., 2011). There are several reasons for this focus, but the most impactful is the fact that fMRI data exhibit substantial signal drop-off as distance increases from the MR coil, particularly with simultaneous multislice (or multiband) acquisitions (Srirangarajan et al., 2021). The result is that fMRI signals in subcortical structures tend to be noisy and have lower amplitude. As such, functional connectivity with known cortical networks is low, and network detection approaches struggle to label subcortical voxels. Specific to fMRI, the way that BOLD signal relates to neural activity varies considerably between cortical and subcortical regions. In the cerebellum, for example (Vaishnavi et al., 2010), Purkinje cells produce weaker changes in the blood flow (Thomsen et al., 2004, 2009) than the neocortex. While it has been difficult to link subcortical regions to cortical networks for these reasons, recent work has made considerable progress on this front, particularly with high field 7T MRI (Fritz et al., 2019; Li et al., 2021).

This methods-driven cortical bias risks ignoring major portions of the brain’s large-scale network architecture. Anatomical tracing studies demonstrate that the major subcortical structures exert critical influence over the cortex via reciprocal or looped circuits. Cortex and cerebellum communicate via the cortico-ponto-cerebellar pathway, which then feeds back to cortex via thalamus (Ramnani, 2006). Separately, cortex, striatum, and thalamus are linked in cortico-striato-thalamo-cortical loops (Alexander et al., 1986). Primate (both nonhuman and human) research shows that these projections, while organized in a general topographic manner on the basis of cortical origin, contain complex interfaces between terminal fields from diverse cortical areas, which allows transfer of information across functional domains (Averbeck et al., 2014; Draganski et al., 2008; Haber, 2010). This suggests that cortical networks will also serve as an organizing principle for subcortical structures (or vice versa). Indeed, specialized network identification approaches that account for low signal do find topographically organized networks in striatum (Choi et al., 2012; Di Martino et al., 2008; Gordon et al., 2022; Greene et al., 2014, 2020; Jarbo & Verstynen, 2015), cerebellum (Buckner et al., 2011; King et al., 2019; Marek et al., 2018; Xue et al., 2021), thalamus (Greene et al., 2020), hippocampus (Zheng et al., 2021), amygdala (Sylvester et al., 2020), and basal forebrain (Markello et al., 2018; Yuan et al., 2019).

In many cases, this network organization converges closely with known anatomical projections in nonhuman primates. For example, cortical somatomotor networks are represented with a topographically preserved organization in posterior putamen (Choi et al., 2012), ventral lateral thalamus (Greene et al., 2020), and both anterior and posterior cerebellar lobes (Buckner et al., 2011). The occipital network has little representation in striatum or cerebellum, but is present in a posterior lateral thalamus region converging with the lateral geniculate nucleus (Greene et al., 2020).

**Figure 6.**
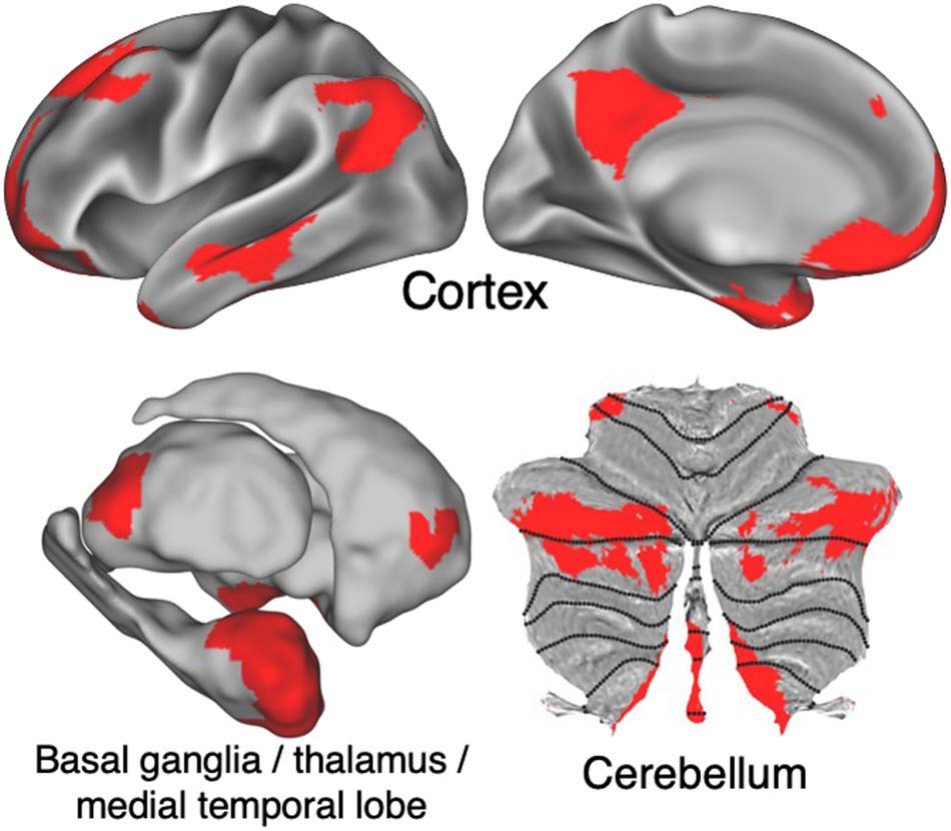
Cortical and subcortical elements of the default network. Anatomical locations of the default network (red) in lateral and medial cortex (top), basal ganglia, thalamus, and medial temporal lobe (bottom left), and cerebellum (bottom right).

In other cases, the subcortical representation of cortical networks that are dramatically altered and expanded in humans relative to other mammals provides novel insight into their organization. While the default network is well known to be represented in a variety of frontal, parietal, and temporal cortical regions, it also has representation in the hippocampus and amygdala (Sylvester et al., 2020; Zheng et al., 2021), ventral striatum (Choi et al., 2012; Gordon et al., 2020, 2022), medial nuclei of the thalamus (Greene et al., 2020), basal forebrain (Markello et al., 2018; Yuan et al., 2019), and at the border between cerebellar Crus I and II (Buckner et al., 2011) (Figure 6). Recent work has begun to map subcortical connectivity of the default network using high-resolution functional imaging (Li et al., 2021).

An accurate taxonomy of networks is incomplete without consideration and inclusion of these subcortical elements. For example, the default network has known roles in processing reward, memory, and emotion (Andrews-Hanna et al., 2014) These functions are incompletely understood without the topography of the default network including subcortical counterparts such as ventral striatum, hippocampus, and amygdala. Furthermore, it is important to note that both anatomic and fMRI studies show not only segregation of subcortical projections based on cortical origin, but also substantial integration and overlap. Taking these aspects into account is thus crucial for better explaining function and behavior based on anatomy. A full review of subcortical affiliations with large-scale cortical networks is beyond the scope of the present work, and substantial work remains to reliably delineate these associations. In this way, any network taxonomy must continue to evolve as new discoveries regarding cortico-subcortical interactions are made.

### Contributions of Multimodal Information

Large-scale brain networks were historically identified by cognitive neurology (Mesulam, 1998) and complemented by comparative neuroanatomical fiber tract tracing (Pandya & Yeterian, 1985). In the last 20 years, fMRI has largely superseded this work, and resting-state functional connectivity has come to dominate investigations of large-scale brain networks. However, electrophysiological imaging modalities have been increasingly utilized in the study of functional networks. In parallel, structural networks have been probed with dMRI tractography. Several studies have compared these techniques, with the goal of investigating the concordance between functional networks derived from fMRI and electrophysiology, or the extent to which these functional networks can be explained by the structural connections derived from dMRI. As we acknowledged at the outset of this project, future work must incorporate findings derived from multiple neuroimaging modalities to enrich our understanding of large-scale brain network taxonomies.

Here we discuss points of multimodal convergence and areas where complementary evidence can be derived from modalities other than fMRI to further the goals of standardizing large-scale brain network nomenclature. Specifically, spatial convergence of large-scale brain networks in structural and other functional data modalities with those observed in fMRI would support the viewpoint that these networks are a fundamental and timescale-overarching organizational principle of the brain (that fMRI is particularly well-poised to measure). As we will detail below, partial cross-modal convergence is indeed observed, thus countering the alternative possibility that large-scale brain networks are unique to fMRI, either due to nonneural signal confounds or confined to a limited temporal bandwidth. Beyond methodological limitations, large-scale brain networks measured by different data modalities may differ in terms of underlying neural organization. Future work into systematic differences is needed to understand whether and how large-scale brain networks spatially vary across connectivity timescales (infraslow in fMRI and fast in EEG/MEG) and across functional and structural connectivity measures. Systematic investigations, building on those discussed below, are crucial to determine whether a modality-overarching network nomenclature, of the kind that is currently used across fMRI, neurophysiological, and structural literature, is productive.

Functional connectivity from fMRI has been compared with electrocorticography (ECoG)/intracranial electroencephalography (iEEG) to verify a neuronal basis for the spatial topography of large-scale brain networks. Spatial convergence between fMRI and intracranial electrophysiology has been robustly observed (Bright et al., 2020; He et al., 2008; Kucyi et al., 2018; Nir et al., 2008). A major goal of early spatial comparisons of fMRI to *scalp recorded* EEG/MEG was to demonstrate the capability of (source-localized) noninvasive electrophysiology to study large-scale brain networks. In particular, large-scale brain networks akin to the “canonical” networks known from fMRI have been observed in scalp recordings (Brookes et al., 2011; de Pasquale et al., 2010; for review see Sadaghiani & Wirsich, 2020). With the reliability of large-scale brain networks largely confirmed, the field can now increasingly focus on the complementary but distinct neurobiological information about large-scale brain networks provided by these different neuroimaging modalities.

Noninvasive scalp EEG/MEG is sufficiently informative to permit the study of large-scale brain networks, yet the necessity of mathematically ill-posed source localization and residual source leakage render these methods spatially less reliable and less resolved than fMRI. On the other hand, invasive ECoG/iEEG provides local field potentials/multiunit activity data on connectivity without providing ‘whole-brain’ data (note, however, pooling electrode pairs over a large number of patients may overcome this issue; Betzel et al., 2019). Counterbalancing these spatial weaknesses, the core strength of electrophysiological methods compared with fMRI is that they allow the study of networks at a finer temporal scale, permitting analysis of their time-varying dynamics (Veit et al., 2021).

The spatial correspondence of whole-brain connectomes between fMRI and electrophysiological methods is significant, but the effect size is typically moderate. This observation holds true irrespective of data modalities (fMRI-to-scalpEEG, fMRI-to-ECoG, fMRI-to-MEG) and methodological and analytic choices (Wirsich et al., 2021). The electrophysiological and hemodynamic connectomes may therefore reflect partially nonoverlapping neural populations (Hari & Parkkonen, 2015). The above-described studies were conducted using resting-state fMRI data. Task-evoked changes relative to resting state have also been explored (Favaretto et al., 2021). An open question is therefore whether the spatial deviations between functional data modalities are systematic. We expect a systematic difference in precise source locations.

Several studies have investigated the structural basis for fMRI-derived large-scale brain networks (see Suárez et al., 2020, for review). Early work derived structural connections either from prior tracer studies in macaques (Honey et al., 2007) or from dMRI tractography in humans (Deco et al., 2013; Goñi et al., 2014; Honey et al., 2009), and simulated functional time courses given these structural connections and random fluctuations in neuronal activity. The comparison of these simulated time courses to those measured empirically by resting-state fMRI showed evidence that functional connectivity may indeed arise from spontaneous activity across regions that are connected structurally. However, direct correlation between edge weights of structural and functional networks is in the low to moderate range (Goñi et al., 2014; Hagmann et al., 2008; Honey et al., 2009). While structural connections can be used to predict functional connectivity (Goñi et al., 2014; Honey et al., 2009; Rosenthal et al., 2018; Sarwar et al., 2021), the reverse is not necessarily true (Honey et al., 2009; but see Wirsich et al., 2017). This has been attributed to the fact that two regions can be coupled functionally even in the absence of a direct structural link between them, if they are linked indirectly via a third region. Several studies have shown that indirect structural connections can predict functional connectivity, although their predictive power is somewhat lower than that of direct structural connections (Goñi et al., 2014; Honey et al., 2009; Rosenthal et al., 2018). The correspondence of structure and function are greatest in unimodal, primary sensory, and motor regions, but diverges in transmodal cortex, particularly among default and salience network regions (Vázquez-Rodríguez et al., 2019). The complex relationships between structural and functional connectivity are highlighted in case studies such as split-brain patients, in whom the cerebral commissures have been disconnected (Uddin, 2013). In the absence of direct interhemispheric structural connections, these patients can still exhibit strong functional connectivity across the hemispheres that is most likely mediated by indirect subcortical pathways (Nomi et al., 2019; Uddin et al., 2008b).

Given the plethora of approaches to dMRI tractography, it is worth considering how algorithmic choices that affect structural connectivity measures obtained from dMRI may impact these findings. Whole-brain structural network analyses have typically utilized deterministic tractography. Validation studies that have compared dMRI tractography to anatomic tracing have shown that, when compared at the same false positive rate, probabilistic tractography methods have higher true positive rates (or, equivalently, lower false negative rates) than deterministic tractography methods (Delettre et al., 2019; Girard et al., 2020; Grisot et al., 2021; Maffei et al., 2022). However, the default thresholds typically used in tractography tend to be conservative. That is, they correspond to low-false-positive, low-true-positive operating points (Grisot et al., 2021). In that regime, any performance differences between probabilistic and deterministic methods are small. Importantly, tractography methods detect the larger structural connections from each region, but miss smaller ones. This is likely to have had an impact on any prior comparisons of structural and functional brain networks, and merits further investigation.

Finally, the relationship between structural and functional networks may vary between functional states (Fukushima et al., 2018; Liu et al., 2022) and with development (Supekar et al., 2010; Uddin et al., 2011). While functional connectivity is of a highly dynamic nature (see section Time-Varying Properties: Dynamics, Nonstationarity, and Contextual Effects on Network Organization), the brain is not rewired structurally at the same rate. This implies that we cannot expect full agreement between functional and structural connectomes. Thus, even after resolving all methodological issues, fMRI and dMRI will provide complementary information about large-scale brain networks. As discussed above, however, the literature does provide evidence for a link between the two, and in particular for (time-averaged) resting-state fMRI networks emerging from correlations of spontaneous activity between regions that are connected structurally. The brief overview of multimodal neuroimaging findings relevant to the goal of standardizing network nomenclature points to many open questions that we hope to see addressed in future iterations of guidelines for developing network taxonomies.

### Interim Conclusion

In this section, we reviewed five significant issues that directly impact the formulation of a universal taxonomy of large-scale brain networks. These issues included the spatial scale and hierarchical organization of networks, interindividual variability, brain dynamics, the consideration of subcortical structures, and multimodal evidence. It is important to emphasize that each of these areas represent ongoing programs of research from multiple labs, including members of WHATNET. Given the plurality of ongoing discoveries necessary to arrive at consensus, and the multitude of plausible solutions given the existing evidence, a universal taxonomy could not be agreed upon at the time of writing this report. In light of these issues, we do not provide concrete recommendations for large-scale brain network nomenclature. However, there was broad agreement on reporting guidelines and avenues for future research to conduct in order to more efficiently integrate current and future findings together toward a broader consensus of large-scale brain network topography.

## TOWARD MINIMAL REPORTING GUIDELINES FOR NETWORK RESULTS

The original COBIDAS report included recommendations and a checklist for sharing statistical maps and for reporting functional connectivity results (Nichols et al., 2017). Specifically, the guidelines suggested that for ICA results, researchers should report the total number of components analyzed, and the rationale for their selection. For graph analyses, the recommendation was to state the null hypothesis of the test and how the statistic distribution under the null was computed. We concur that these are important pieces of information to include in the results sections of manuscripts. As we have discussed throughout, coming up with a complete checklist of reporting guidelines similar to that in the original COBIDAS report that is specific for network neuroscience results is no simple task. Here we summarize some points of consensus among WHATNET members regarding best practices for reporting results from studies in which large-scale brain networks are investigated (Box 1).

**Box 1.** Recommendations for reporting network results.

1)  Task fMRI contrasts derived from univariate GLM analysis do not necessarily comprise a network
2)  Avoid labeling patterns of brain activity or connectivity with only an idiosyncratic cognitive term
3)  To determine network affiliations of novel findings, use and reference one or more existing parcellation schemes (Kong et al., 2022)
4)  Report sample variation from the population used to generate the reference parcellation scheme
5)  Consider supplementing atlas labels with additional anatomical network labels (such as those proposed in Uddin et al. (2019) for ease of integration across studies
6)  Follow COBIDAS reporting guidelines (Nichols et al., 2017) for connectivity analysis

There is a growing use of network-based approaches to identify large-scale brain networks from task fMRI data in cognitive neuroscience. Researchers often compute functional connectivity from task fMRI data to reveal how large-scale brain networks respond to experimental manipulation (Metzak et al., 2011). Still, one point for researchers conducting task-based fMRI to keep in mind is that the results of a univariate general linear model (GLM) contrast between two cognitive conditions does not necessarily equate to a network, however tempting it may be to use network nomenclature to describe activation results when they spatially resemble other large-scale brain networks that have been described in the literature. One suggestion from this group is to avoid giving descriptive cognitive names to networks, particularly when describing idiosyncratic cognitive domains (e.g., reward network, pain network). In this way, we can avoid proliferation of network naming terminologies and more readily compare results across studies. For example, large-scale brain networks occupying the territory of the lateral fronto-parietal area have been variably referred to as the central executive or executive control network (Seeley et al., 2007), the multiple-demand system (Duncan, 2010), the extrinsic mode network (Hugdahl et al., 2015), the domain general system (Fedorenko et al., 2013), the fronto-parietal control network (Dosenbach et al., 2008; Vincent et al., 2008), and the cognitive control network (Niendam et al., 2012). Our own survey showed that these networks were among the least agreed upon among independent raters. We suggest that naming networks by a single purported cognitive function is antithetical to the goal of understanding the broad role large-scale brain networks play in cognition, and hinders the development of a universal taxonomy.

**Figure 7.**
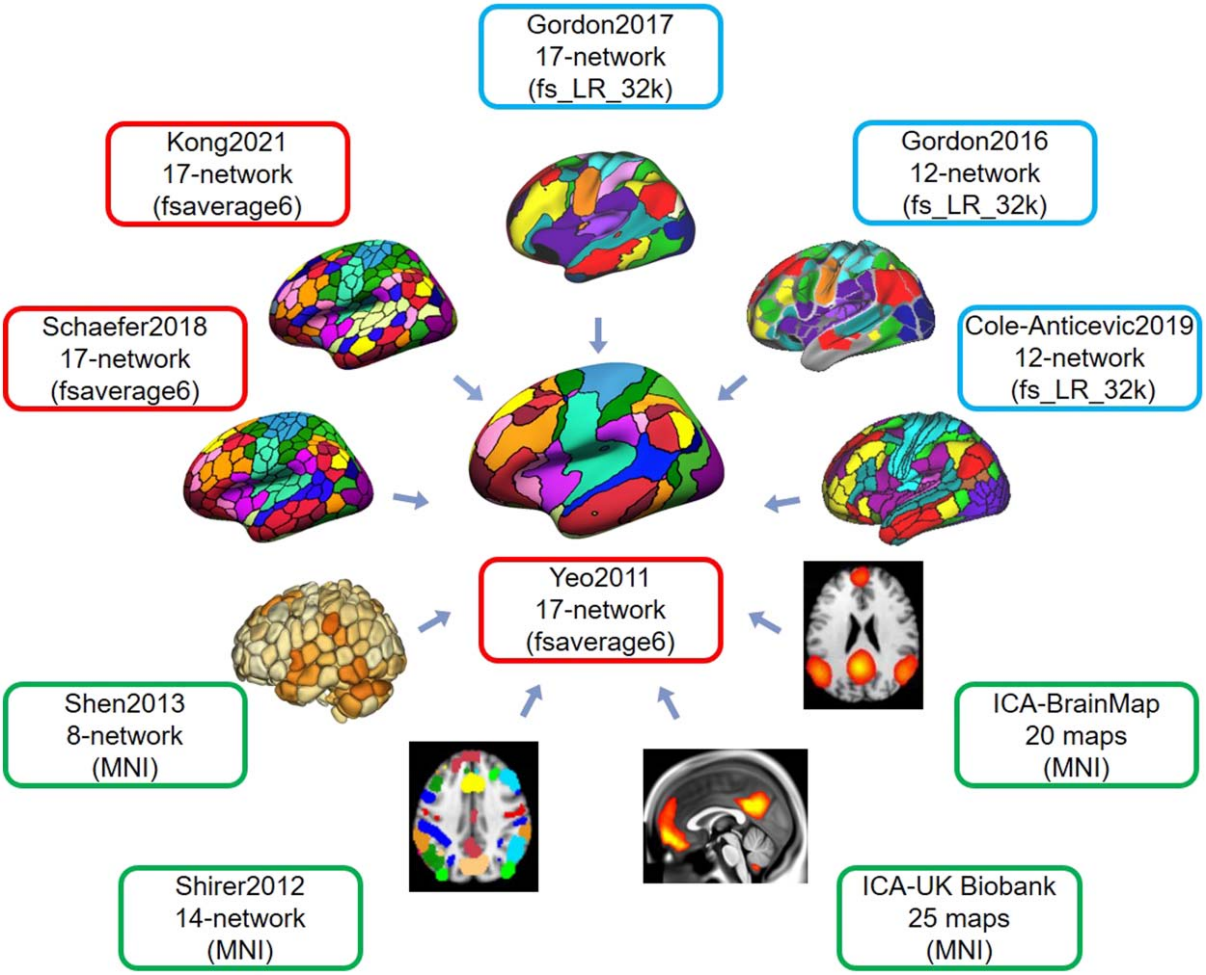
Ten representative group-level functional brain network atlases. In this example, Yeo’s 17-network atlas serves as the reference atlas, and all other atlases are projected to the same space to compute overlap with the reference network (from Kong et al., 2022).

The suggestion instead would be to evaluate any new findings, whether in the task fMRI or resting-state fMRI domain, against one or more commonly used parcellation schemes. This recommendation extends to large-scale connectivity in electrophysiological data, in spite of the fact that the currently common parcellation schemes are derived from fMRI. Some tools are already available to aid users in automatically labeling results of novel analyses with the aid of multiple atlases (Fang et al., 2019; Salman et al., 2022). Given a previously published parcellation and a set of functional maps, one can determine the extent to which a novel functional map overlaps with a predefined atlas (Kong et al., 2022) (Figure 7). In doing so, we suggest that one clearly state which reference atlas is being utilized, and whether the demographic characteristics of the individuals used to make that atlas match the characteristics of the group from which the novel data were obtained. Acknowledgment of potential sources of variability should be openly discussed. Probabilistic atlases (Dworetsky et al., 2021) can in some cases be referenced to note what types of individual differences might be expected, and discuss how this might affect the network designations in any new report. For example, one might exercise more caution in applying the “fronto-parietal network” label than the “visual network,” label given the greater potential variability in the former than in the latter.

Complimentary anatomical labels may be specified alongside functional atlas-based labels in some cases to provide additional information (Uddin et al., 2019). That way, if a new study reports findings relevant to a scholar interested in following research on a given large-scale brain network, the results will be more readily discoverable. Researchers should clearly report which atlas or parcellation scheme was used, and follow the original COBIDAS guideline regarding which space the findings are reported in, as well as the guidelines for sharing raw data and maps.

An additional guideline from this workgroup relates to the discussion on brain dynamics. When defining or describing networks in a particular study, one should consider that large-scale brain networks undergo functionally relevant spatial variations across time and cognitive contexts, and consequently may not fully match standard network parcellations derived from static resting-state fMRI data.

## UNRESOLVED ISSUES AND FUTURE DIRECTIONS

We have aimed to cover a range of literature relevant to the problem of building a universal taxonomy of large-scale brain networks. However, we readily acknowledge that this manuscript should be considered a living document, subject to continuous revision to incorporate new data and theoretical frameworks as they become available. Note that here, we provide recommendations for the types of information that we suggest network neuroscience papers should report going forward. Unlike the 2019 taxonomy proposal (Uddin et al., 2019), however, we do not provide recommendations for specific names and labels to give to large-scale brain networks in future studies. A strictly anatomical labeling scheme is not flexible enough to account for current methodological limitations. Anatomical normalization procedures failed to capture between-subject anatomical variance, which subsequently blur aspects of between-subject functional variability and estimates of brain dynamics that are still open areas of inquiry for the field. Still, we contend that a strictly functional scheme would be insufficient, given the plurality of cognitive functions subserved by nearly every large-scale brain network that has been identified to date.

As alluded to earlier, the field is only beginning to tackle the issue of how best to categorize large-scale brain networks in developmental, aging, and clinical populations. This is particularly problematic given that network coherence changes across human development, with increasing network differentiation in early development (Cui et al., 2020) and reduced segregation in older adults late in life (Han et al., 2018; Setton et al., 2023). This issue has been addressed in the developmental neuroimaging literature using study-specific templates for normalization (Sanchez et al., 2012). One can imagine an analogous scenario in which study-specific parcellations might be appropriate for a specific research question, such as studying a developmental cohort (Hermosillo et al., 2022).

With regards to network variability as observed in clinical populations, several open questions remain. For example, if we see that a portion of a large-scale brain network is missing consistently in a clinical group, does this tell us something about the “core” components of that network? It is not always clear whether differences observed in clinical populations index loss of function, decreased efficiency, or compensatory reorganization processes associated with recovery. A first step may be to use individual-level network definitions (e.g., via data-driven clustering, ICA, or prior-based techniques) to identify brain networks within each group and create group-specific probabilistic atlases (Dworetsky et al., 2023; Hermosillo et al., 2022). These can then be used to guide future studies on the importance of differences in network organization across populations.

We have not yet attempted to consider relevant cross-species comparisons in the current work. There is increasing evidence, for example, that an analog of the human default network can be identified in nonhuman primates (Mantini et al., 2011) and rodents (Lu et al., 2012). Understanding these cross-species convergences may help further delineate large-scale brain network properties in humans by permitting investigation of the degree to which network topographies are evolutionarily conserved (Buckner & Krienen, 2013).

A subset of the WHATNET group is currently working on a tool that will allow users to quantify the spatial overlap between their findings and one (or more) of 16 commonly used parcellation schemes/atlases (Kong et al., 2022). Network overlap across atlases was computed by treating each atlas as the reference, and projecting other atlases to that reference atlas space to compute the Dice coefficient between the reference network and each network from all other atlases. This tool is currently under development for release to the public, and will provide a means for mapping between any given set of new results and one or several widely used brain atlases for reporting purposes. We suggest that this type of atlas-referenced reporting should become the norm for future investigations.

Finally, we suggest that the field of cognitive neuroscience might make rapid progress toward the goals of WHATNET by adopting the practice of adversarial collaboration, whereby investigators committed to different theoretical views collaborate to test opposing predictions. Our survey of the neuroimaging community revealed the least amount of agreement among raters when they were naming networks involving fronto-parietal and midcingulo-insular cortical areas. Interestingly, our tool that is currently under development revealed that the network labeled “salience/ventral attention network A” from the Yeo2011 atlas spatially overlaps to a great extent with the network labeled “cingulo-opercular network” from the Gordon2017 atlas. One suggestion would be for researchers who have coined particular network names such as “salience” (Seeley et al., 2007), “cingulo-opercular” (Dosenbach et al., 2008) and “ventral attention” (Corbetta & Shulman, 2002) to collaborate to design a set of experiments that would engage the putative cognitive functions associated with each of these large-scale brain networks. Collaborative efforts of this type may help resolve ambiguities and inconsistencies going forward. Adversarial collaborations are currently under way in the field of consciousness research, which has for years been fragmented due to multiple theoretical perspectives (Melloni et al., 2021). We envision that well-planned, preregistered cognitive neuroscience investigations that more closely map large-scale brain networks to cognitive processes might help reduce the proliferation of network names going forward.

## ACKNOWLEDGMENTS

The authors would like to thank the OHBM Best Practices committee, Remi Gau, Tom Johnstone, and Matt Glasser for constructive feedback on earlier versions of this manuscript. In addition, the authors would like to thank Trey Boone for assistance with analysis of results from the survey, and Ruby Kong for creating Figure 7.

## AUTHOR CONTRIBUTIONS

Lucina Q. Uddin: Conceptualization; Funding acquisition; Investigation; Methodology; Project administration; Supervision; Writing – original draft; Writing – review & editing. Richard F. Betzel: Conceptualization; Investigation; Methodology; Writing – original draft; Writing – review & editing. Jessica R. Cohen: Conceptualization; Investigation; Methodology; Writing – original draft; Writing – review & editing. Jessica S. Damoiseaux: Conceptualization; Investigation; Methodology; Writing – original draft; Writing – review & editing. Felipe De Brigard: Conceptualization; Data curation; Formal analysis; Visualization; Writing – original draft; Writing – review & editing. Simon B. Eickhoff: Conceptualization; Investigation; Writing – original draft; Writing – review & editing. Alex Fornito: Conceptualization; Investigation; Writing – original draft; Writing – review & editing. Caterina Gratton: Conceptualization; Investigation; Visualization; Writing – original draft; Writing – review & editing. Evan M. Gordon: Conceptualization; Investigation; Visualization; Writing – original draft; Writing – review & editing. Angela R. Laird: Conceptualization; Investigation; Writing – original draft; Writing – review & editing. Linda Larson-Prior: Conceptualization; Data curation; Formal analysis; Investigation; Writing – original draft; Writing – review & editing. A. Randal McIntosh: Conceptualization; Investigation; Writing – original draft; Writing – review & editing. Lisa D. Nickerson: Conceptualization; Data curation; Formal analysis; Investigation; Visualization; Writing – original draft; Writing – review & editing. Luiz Pessoa: Conceptualization; Investigation; Writing – original draft; Writing – review & editing. Ana Luísa Pinho: Conceptualization; Investigation; Writing – original draft; Writing – review & editing. Russell A. Poldrack: Conceptualization; Investigation; Writing – original draft; Writing – review & editing. Adeel Razi: Conceptualization; Investigation; Writing – original draft; Writing – review & editing. Sepideh Sadaghiani: Conceptualization; Investigation; Writing – original draft; Writing – review & editing. James M. Shine: Conceptualization; Investigation; Writing – original draft; Writing – review & editing. Anastasia Yendiki: Conceptualization; Investigation; Writing – original draft; Writing – review & editing. B. T. Thomas Yeo: Conceptualization; Data curation; Formal analysis; Investigation; Visualization; Writing – original draft; Writing – review & editing. R. Nathan Spreng: Conceptualization; Data curation; Formal analysis; Investigation; Supervision; Writing – original draft; Writing – review & editing.

## FUNDING INFORMATION

LP is supported by NIMH (MH071589 and MH112517). AY is supported by NIH R01EB021265, R01NS119911, P50MH106435, and U01EB026996. CG is supported by NSF CAREER 2048066 and NIH R01MH118370. JRC is supported by NIH R01MH119091. FDB is supported by NSF FAIN 2218556. LQU is supported by NIH U01DA050987. SS is supported by NIH R01MH116226 and NIH R21NS104603. ALP is the recipient of a BrainsCAN Postdoctoral Fellowship at Western University, funded by the Canada First Research Excellence Fund (CFREF). RNS is supported by the Canadian Institute of Health Research, Natural Sciences and Engineering Research Council of Canada, NIA R01AG068563, and Fonds de recherche du Québec – Santé.

## TECHNICAL TERMS

Network: A system of elements (nodes, vertices) and their pairwise interactions (edges, connections, links) used to model a diverse array of technological, social, physical, and biological systems.
Graph: A mathematical characterization of a network, comprising nodes (neural elements, in the current context) and edges (the relations between pairs of nodes).
Node: A fundamental element or processing unit of a network.
Edge: A connection (structural or functional) between the nodes of a network.
Large-scale brain network: A group of macroscopic neural elements that show correlated activity. In humans, such networks are typically identified from neuroimaging data using quantitative techniques and modeling frameworks.
Functional brain network: A group of neural elements that show correlated activity, from ensembles of cells to collections of macroscopic brain regions.
Subnetwork: A group of dynamically coherent neural elements that is nested (sometimes hierarchically) within a larger brain network.
Spatial independent component analysis (sICA): A multivariate analytic approach capable of identifying brain networks at multiple spatial scales.

## References

[bib1] Abou-Elseoud, A., Starck, T., Remes, J., Nikkinen, J., Tervonen, O., & Kiviniemi, V. (2010). The effect of model order selection in group PICA. Human Brain Mapping, 31(8), 1207–1216. 10.1002/hbm.20929, 20063361 PMC6871136

[bib2] Alexander, G. E., DeLong, M. R., & Strick, P. L. (1986). Parallel organization of functionally segregated circuits linking basal ganglia and cortex. Annual Review of Neuroscience, 9, 357–381. 10.1146/annurev.ne.09.030186.002041, 3085570

[bib3] Allen, E. A., Damaraju, E., Plis, S. M., Erhardt, E. B., Eichele, T., & Calhoun, V. D. (2014). Tracking whole-brain connectivity dynamics in the resting state. Cerebral Cortex, 24(3), 663–676. 10.1093/cercor/bhs352, 23146964 PMC3920766

[bib4] Anderson, K. M., Ge, T., Kong, R., Patrick, L. M., Spreng, R. N., Sabuncu, M. R., Yeo, B. T. T., & Holmes, A. J. (2021). Heritability of individualized cortical network topography. Proceedings of the National Academy of Sciences of the United States of America, 118(9), e2016271118. 10.1073/pnas.2016271118, 33622790 PMC7936334

[bib5] Andrews-Hanna, J. R., Reidler, J. S., Sepulcre, J., Poulin, R., & Buckner, R. L. (2010). Functional-anatomic fractionation of the brain’s default network. Neuron, 65(4), 550–562. 10.1016/j.neuron.2010.02.005, 20188659 PMC2848443

[bib6] Andrews-Hanna, J. R., Smallwood, J., & Spreng, R. N. (2014). The default network and self-generated thought: Component processes, dynamic control, and clinical relevance. Annals of the New York Academy of Sciences, 1316(1), 29–52. 10.1111/nyas.12360, 24502540 PMC4039623

[bib7] Averbeck, B. B., Lehman, J., Jacobson, M., & Haber, S. N. (2014). Estimates of projection overlap and zones of convergence within frontal-striatal circuits. Journal of Neuroscience, 34(29), 9497–9505. 10.1523/JNEUROSCI.5806-12.2014, 25031393 PMC4099536

[bib8] Bassett, D. S., Wymbs, N. F., Porter, M. A., Mucha, P. J., Carlson, J. M., & Grafton, S. T. (2011). Dynamic reconfiguration of human brain networks during learning. Proceedings of the National Academy of Sciences of the United States of America, 108(18), 7641–7646. 10.1073/pnas.1018985108, 21502525 PMC3088578

[bib9] Bassett, D. S., Yang, M., Wymbs, N. F., & Grafton, S. T. (2015). Learning-induced autonomy of sensorimotor systems. Nature Neuroscience, 18(5), 744–751. 10.1038/nn.3993, 25849989 PMC6368853

[bib10] Bastiani, M., Shah, N. J., Goebel, R., & Roebroeck, A. (2012). Human cortical connectome reconstruction from diffusion weighted MRI: The effect of tractography algorithm. NeuroImage, 62(3), 1732–1749. 10.1016/j.neuroimage.2012.06.002, 22699045

[bib11] Baum, G. L., Roalf, D. R., Cook, P. A., Ciric, R., Rosen, A. F. G., Xia, C., Elliott, M. A., Ruparel, K., Verma, R., Tunç, B., Gur, R. C., Gur, R. E., Bassett, D. S., & Satterthwaite, T. D. (2018). The impact of in-scanner head motion on structural connectivity derived from diffusion MRI. NeuroImage, 173, 275–286. 10.1016/j.neuroimage.2018.02.041, 29486323 PMC5911236

[bib12] Beckmann, C. F., DeLuca, M., Devlin, J. T., & Smith, S. M. (2005). Investigations into resting-state connectivity using independent component analysis. Philosophical Transactions of the Royal Society of London Series B: Biological Sciences, 360(1457), 1001–1013. 10.1098/rstb.2005.1634, 16087444 PMC1854918

[bib13] Bellec, P., Rosa-Neto, P., Lyttelton, O. C., Benali, H., & Evans, A. C. (2010). Multi-level bootstrap analysis of stable clusters in resting-state fMRI. NeuroImage, 51(3), 1126–1139. 10.1016/j.neuroimage.2010.02.082, 20226257

[bib14] Bertolero, M. A., Yeo, B. T. T., & D’Esposito, M. (2015). The modular and integrative functional architecture of the human brain. Proceedings of the National Academy of Sciences of the United States of America, 112(49), E6798–E6807. 10.1073/pnas.1510619112, 26598686 PMC4679040

[bib15] Betzel, R. F., & Bassett, D. S. (2017). Multi-scale brain networks. NeuroImage, 160, 73–83. 10.1016/j.neuroimage.2016.11.006, 27845257 PMC5695236

[bib16] Betzel, R. F., Cutts, S. A., Greenwell, S., Faskowitz, J., & Sporns, O. (2022). Individualized event structure drives individual differences in whole-brain functional connectivity. NeuroImage, 252, 118993. 10.1016/j.neuroimage.2022.118993, 35192942

[bib17] Betzel, R. F., Fukushima, M., He, Y., Zuo, X.-N., & Sporns, O. (2016). Dynamic fluctuations coincide with periods of high and low modularity in resting-state functional brain networks. NeuroImage, 127, 287–297. 10.1016/j.neuroimage.2015.12.001, 26687667 PMC4755785

[bib18] Betzel, R. F., Medaglia, J. D., Kahn, A. E., Soffer, J., Schonhaut, D. R., & Bassett, D. S. (2019). Structural, geometric and genetic factors predict interregional brain connectivity patterns probed by electrocorticography. Nature Biomedical Engineering, 3(11), 902–916. 10.1038/s41551-019-0404-5, 31133741

[bib19] Bijsterbosch, J. D., Beckmann, C. F., Woolrich, M. W., Smith, S. M., & Harrison, S. J. (2019). The relationship between spatial configuration and functional connectivity of brain regions revisited. eLife, 8, e44890. 10.7554/eLife.44890, 31066676 PMC6541435

[bib20] Bijsterbosch, J. D., Harrison, S. J., Jbabdi, S., Woolrich, M., Beckmann, C., Smith, S., & Duff, E. P. (2020). Challenges and future directions for representations of functional brain organization. Nature Neuroscience, 23(12), 1484–1495. 10.1038/s41593-020-00726-z, 33106677

[bib21] Bijsterbosch, J. D., Woolrich, M. W., Glasser, M. F., Robinson, E. C., Beckmann, C. F., Van Essen, D. C., Harrison, S. J., & Smith, S. M. (2018). The relationship between spatial configuration and functional connectivity of brain regions. eLife, 7, e32992. 10.7554/eLife.32992, 29451491 PMC5860869

[bib22] Birn, R. M., Smith, M. A., Jones, T. B., & Bandettini, P. A. (2008). The respiration response function: The temporal dynamics of fMRI signal fluctuations related to changes in respiration. NeuroImage, 40(2), 644–654. 10.1016/j.neuroimage.2007.11.059, 18234517 PMC2533266

[bib23] Biswal, B., Yetkin, F. Z., Haughton, V. M., & Hyde, J. S. (1995). Functional connectivity in the motor cortex of resting human brain using echo-planar MRI. Magnetic Resonance in Medicine, 34(4), 537–541. 10.1002/mrm.1910340409, 8524021

[bib550] Bolt, T., Nomi, J. S., Rubinov, M., & Uddin, L. Q. (2017). Correspondence between evoked and intrinsic functional brain network configurations. Human Brain Mapping, 38(4), 1992–2007. 10.1002/hbm.23500, 28052450 PMC6866760

[bib24] Braga, R. M., & Buckner, R. L. (2017). Parallel interdigitated distributed networks within the individual estimated by intrinsic functional connectivity. Neuron, 95(2), 457–471. 10.1016/j.neuron.2017.06.038, 28728026 PMC5519493

[bib25] Braga, R. M., DiNicola, L. M., Becker, H. C., & Buckner, R. L. (2020). Situating the left-lateralized language network in the broader organization of multiple specialized large-scale distributed networks. Journal of Neurophysiology, 124(5), 1415–1448. 10.1152/jn.00753.2019, 32965153 PMC8356783

[bib26] Braga, R. M., Van Dijk, K. R. A., Polimeni, J. R., Eldaief, M. C., & Buckner, R. L. (2019). Parallel distributed networks resolved at high resolution reveal close juxtaposition of distinct regions. Journal of Neurophysiology, 121(4), 1513–1534. 10.1152/jn.00808.2018, 30785825 PMC6485740

[bib27] Braun, U., Schäfer, A., Walter, H., Erk, S., Romanczuk-Seiferth, N., Haddad, L., Schweiger, J. I., Grimm, O., Heinz, A., Tost, H., Meyer-Lindenberg, A., & Bassett, D. S. (2015). Dynamic reconfiguration of frontal brain networks during executive cognition in humans. Proceedings of the National Academy of Sciences of the United States of America, 112(37), 11678–11683. 10.1073/pnas.1422487112, 26324898 PMC4577153

[bib28] Bright, M. G., Whittaker, J. R., Driver, I. D., & Murphy, K. (2020). Vascular physiology drives functional brain networks. NeuroImage, 217, 116907. 10.1016/j.neuroimage.2020.116907, 32387624 PMC7339138

[bib29] Brodmann, K. (1909). Vergleichende Lokalisationslehre der Grosshirnrinde in ihren Prinzipien dargestellt auf Grund des Zellenbaues. Barth.

[bib30] Brookes, M. J., Woolrich, M., Luckhoo, H., Price, D., Hale, J. R., Stephenson, M. C., Barnes, G. R., Smith, S. M., & Morris, P. G. (2011). Investigating the electrophysiological basis of resting state networks using magnetoencephalography. Proceedings of the National Academy of Sciences of the United States of America, 108(40), 16783–16788. 10.1073/pnas.1112685108, 21930901 PMC3189080

[bib31] Buckner, R. L., Andrews-Hanna, J. R., & Schacter, D. L. (2008). The brain’s default network: Anatomy, function, and relevance to disease. Annals of the New York Academy of Sciences, 1124, 1–38. 10.1196/annals.1440.011, 18400922

[bib32] Buckner, R. L., & DiNicola, L. M. (2019). The brain’s default network: Updated anatomy, physiology and evolving insights. Nature Reviews Neuroscience, 20(10), 593–608. 10.1038/s41583-019-0212-7, 31492945

[bib33] Buckner, R. L., & Krienen, F. M. (2013). The evolution of distributed association networks in the human brain. Trends in Cognitive Sciences, 17(12), 648–665. 10.1016/j.tics.2013.09.017, 24210963

[bib34] Buckner, R. L., Krienen, F. M., Castellanos, A., Diaz, J. C., & Yeo, B. T. T. (2011). The organization of the human cerebellum estimated by intrinsic functional connectivity. Journal of Neurophysiology, 106(5), 2322–2345. 10.1152/jn.00339.2011, 21795627 PMC3214121

[bib35] Bullmore, E., & Sporns, O. (2009). Complex brain networks: Graph theoretical analysis of structural and functional systems. Nature Reviews Neuroscience, 10(3), 186–198. 10.1038/nrn2575, 19190637

[bib36] Bullmore, E. T., Frangou, S., & Murray, R. M. (1997). The dysplastic net hypothesis: An integration of developmental and dysconnectivity theories of schizophrenia. Schizophrenia Research, 28(2–3), 143–156. 10.1016/S0920-9964(97)00114-X, 9468349

[bib37] Burgess, G. C., Kandala, S., Nolan, D., Laumann, T. O., Power, J. D., Adeyemo, B., Harms, M. P., Petersen, S. E., & Barch, D. M. (2016). Evaluation of denoising strategies to address motion-correlated artifacts in resting-state functional magnetic resonance imaging data from the Human Connectome Project. Brain Connectivity, 6(9), 669–680. 10.1089/brain.2016.0435, 27571276 PMC5105353

[bib38] Calhoun, V. D., Adali, T., Pearlson, G. D., & Pekar, J. J. (2001). A method for making group inferences from functional MRI data using independent component analysis. Human Brain Mapping, 14(3), 140–151. 10.1002/hbm.1048, 11559959 PMC6871952

[bib39] Calhoun, V. D., Miller, R., Pearlson, G., & Adalı, T. (2014). The chronnectome: Time-varying connectivity networks as the next frontier in fMRI data discovery. Neuron, 84(2), 262–274. 10.1016/j.neuron.2014.10.015, 25374354 PMC4372723

[bib40] Chang, C., & Glover, G. H. (2009). Relationship between respiration, end-tidal CO_2_, and BOLD signals in resting-state fMRI. NeuroImage, 47(4), 1381–1393. 10.1016/j.neuroimage.2009.04.048, 19393322 PMC2721281

[bib41] Chang, C., & Glover, G. H. (2010). Time-frequency dynamics of resting-state brain connectivity measured with fMRI. NeuroImage, 50(1), 81–98. 10.1016/j.neuroimage.2009.12.011, 20006716 PMC2827259

[bib42] Choi, E. Y., Yeo, B. T. T., & Buckner, R. L. (2012). The organization of the human striatum estimated by intrinsic functional connectivity. Journal of Neurophysiology, 108(8), 2242–2263. 10.1152/jn.00270.2012, 22832566 PMC3545026

[bib43] Chong, M., Bhushan, C., Joshi, A. A., Choi, S., Haldar, J. P., Shattuck, D. W., Spreng, R. N., & Leahy, R. M. (2017). Individual parcellation of resting fMRI with a group functional connectivity prior. NeuroImage, 156, 87–100. 10.1016/j.neuroimage.2017.04.054, 28478226 PMC5774339

[bib44] Churchland, P. S., & Sejnowski, T. J. (1988). Perspectives on cognitive neuroscience. Science, 242(4879), 741–745. 10.1126/science.3055294, 3055294

[bib45] Ciric, R., Wolf, D. H., Power, J. D., Roalf, D. R., Baum, G. L., Ruparel, K., Shinohara, R. T., Elliott, M. A., Eickhoff, S. B., Davatzikos, C., Gur, R. C., Gur, R. E., Bassett, D. S., & Satterthwaite, T. D. (2017). Benchmarking of participant-level confound regression strategies for the control of motion artifact in studies of functional connectivity. NeuroImage, 154, 174–187. 10.1016/j.neuroimage.2017.03.020, 28302591 PMC5483393

[bib46] Cocchi, L., Halford, G. S., Zalesky, A., Harding, I. H., Ramm, B. J., Cutmore, T., Shum, D. H. K., & Mattingley, J. B. (2014). Complexity in relational processing predicts changes in functional brain network dynamics. Cerebral Cortex, 24(9), 2283–2296. 10.1093/cercor/bht075, 23563963

[bib47] Cocuzza, C. V., Ito, T., Schultz, D., Bassett, D. S., & Cole, M. W. (2020). Flexible coordinator and switcher hubs for adaptive task control. Journal of Neuroscience, 40(36), 6949–6968. 10.1523/JNEUROSCI.2559-19.2020, 32732324 PMC7470914

[bib551] Cohen, J. R. (2018). The behavioral and cognitive relevance of time-varying, dynamic changes in functional connectivity. NeuroImage, 180(Pt B), 515–525. 10.1016/j.neuroimage.2017.09.036, 28942061 PMC6056319

[bib48] Cohen, J. R., & D’Esposito, M. (2016). The segregation and integration of distinct brain networks and their relationship to cognition. Journal of Neuroscience, 36(48), 12083–12094. 10.1523/JNEUROSCI.2965-15.2016, 27903719 PMC5148214

[bib49] Cole, M. W., Bassett, D. S., Power, J. D., Braver, T. S., & Petersen, S. E. (2014). Intrinsic and task-evoked network architectures of the human brain. Neuron, 83(1), 238–251. 10.1016/j.neuron.2014.05.014, 24991964 PMC4082806

[bib50] Cole, M. W., Reynolds, J. R., Power, J. D., Repovs, G., Anticevic, A., & Braver, T. S. (2013). Multi-task connectivity reveals flexible hubs for adaptive task control. Nature Neuroscience, 16(9), 1348–1355. 10.1038/nn.3470, 23892552 PMC3758404

[bib51] Corbetta, M., & Shulman, G. L. (2002). Control of goal-directed and stimulus-driven attention in the brain. Nature Reviews Neuroscience, 3(3), 201–215. 10.1038/nrn755, 11994752

[bib52] Craddock, R. C., James, G. A., Holtzheimer, P. E., 3rd, Hu, X. P., & Mayberg, H. S. (2012). A whole brain fMRI atlas generated via spatially constrained spectral clustering. Human Brain Mapping, 33(8), 1914–1928. 10.1002/hbm.21333, 21769991 PMC3838923

[bib53] Cui, Z., Li, H., Xia, C. H., Larsen, B., Adebimpe, A., Baum, G. L., Cieslak, M., Gur, R. E., Gur, R. C., Moore, T. M., Oathes, D. J., Alexander-Bloch, A. F., Raznahan, A., Roalf, D. R., Shinohara, R. T., Wolf, D. H., Davatzikos, C., Bassett, D. S., Fair, D. A., … Satterthwaite, T. D. (2020). Individual variation in functional topography of association networks in youth. Neuron, 106(2), 340–353. 10.1016/j.neuron.2020.01.029, 32078800 PMC7182484

[bib54] Damoiseaux, J. S. (2017). Effects of aging on functional and structural brain connectivity. NeuroImage, 160, 32–40. 10.1016/j.neuroimage.2017.01.077, 28159687

[bib55] Damoiseaux, J. S., Beckmann, C. F., Arigita, E. J. S., Barkhof, F., Scheltens, P., Stam, C. J., Smith, S. M., & Rombouts, S. A. R. B. (2008). Reduced resting-state brain activity in the “default network” in normal aging. Cerebral Cortex, 18(8), 1856–1864. 10.1093/cercor/bhm207, 18063564

[bib56] Damoiseaux, J. S., Prater, K. E., Miller, B. L., & Greicius, M. D. (2012). Functional connectivity tracks clinical deterioration in Alzheimer’s disease. Neurobiology of Aging, 33(4), 828.e19–828.e30. 10.1016/j.neurobiolaging.2011.06.024, 21840627 PMC3218226

[bib57] Deco, G., Ponce-Alvarez, A., Mantini, D., Romani, G. L., Hagmann, P., & Corbetta, M. (2013). Resting-state functional connectivity emerges from structurally and dynamically shaped slow linear fluctuations. Journal of Neuroscience, 33(27), 11239–11252. 10.1523/JNEUROSCI.1091-13.2013, 23825427 PMC3718368

[bib58] Delbeuck, X., Van der Linden, M., & Collette, F. (2003). Alzheimer’s disease as a disconnection syndrome? Neuropsychology Review, 13(2), 79–92. 10.1023/A:1023832305702, 12887040

[bib59] Delettre, C., Messé, A., Dell, L.-A., Foubet, O., Heuer, K., Larrat, B., Meriaux, S., Mangin, J.-F., Reillo, I., de Juan Romero, C., Borrell, V., Toro, R., & Hilgetag, C. C. (2019). Comparison between diffusion MRI tractography and histological tract-tracing of cortico-cortical structural connectivity in the ferret brain. Network Neuroscience, 3(4), 1038–1050. 10.1162/netn_a_00098, 31637337 PMC6777980

[bib60] de Pasquale, F., Della Penna, S., Snyder, A. Z., Lewis, C., Mantini, D., Marzetti, L., Belardinelli, P., Ciancetta, L., Pizzella, V., Romani, G. L., & Corbetta, M. (2010). Temporal dynamics of spontaneous MEG activity in brain networks. Proceedings of the National Academy of Sciences of the United States of America, 107(13), 6040–6045. 10.1073/pnas.0913863107, 20304792 PMC2851876

[bib61] Desikan, R. S., Ségonne, F., Fischl, B., Quinn, B. T., Dickerson, B. C., Blacker, D., Buckner, R. L., Dale, A. M., Maguire, R. P., Hyman, B. T., Albert, M. S., & Killiany, R. J. (2006). An automated labeling system for subdividing the human cerebral cortex on MRI scans into gyral based regions of interest. NeuroImage, 31(3), 968–980. 10.1016/j.neuroimage.2006.01.021, 16530430

[bib62] Di Martino, A., Scheres, A., Margulies, D. S., Kelly, A. M. C., Uddin, L. Q., Shehzad, Z., Biswal, B., Walters, J. R., Castellanos, F. X., & Milham, M. P. (2008). Functional connectivity of human striatum: A resting state fMRI study. Cerebral Cortex, 18(12), 2735–2747. 10.1093/cercor/bhn041, 18400794

[bib63] DiNicola, L. M., Braga, R. M., & Buckner, R. L. (2020). Parallel distributed networks dissociate episodic and social functions within the individual. Journal of Neurophysiology, 123(3), 1144–1179. 10.1152/jn.00529.2019, 32049593 PMC7099479

[bib64] Dixon, M. L., Andrews-Hanna, J. R., Spreng, R. N., Irving, Z. C., Mills, C., Girn, M., & Christoff, K. (2017). Interactions between the default network and dorsal attention network vary across default subsystems, time, and cognitive states. NeuroImage, 147, 632–649. 10.1016/j.neuroimage.2016.12.073, 28040543

[bib65] Dixon, M. L., De La Vega, A., Mills, C., Andrews-Hanna, J., Spreng, R. N., Cole, M. W., & Christoff, K. (2018). Heterogeneity within the frontoparietal control network and its relationship to the default and dorsal attention networks. Proceedings of the National Academy of Sciences of the United States of America, 115(7), E1598–E1607. 10.1073/pnas.1715766115, 29382744 PMC5816169

[bib66] Donahue, C. J., Sotiropoulos, S. N., Jbabdi, S., Hernandez-Fernandez, M., Behrens, T. E., Dyrby, T. B., Coalson, T., Kennedy, H., Knoblauch, K., Van Essen, D. C., & Glasser, M. F. (2016). Using diffusion tractography to predict cortical connection strength and distance: A quantitative comparison with tracers in the monkey. Journal of Neuroscience, 36(25), 6758–6770. 10.1523/JNEUROSCI.0493-16.2016, 27335406 PMC4916250

[bib67] Dosenbach, N. U. F., Fair, D. A., Cohen, A. L., Schlaggar, B. L., & Petersen, S. E. (2008). A dual-networks architecture of top-down control. Trends in Cognitive Sciences, 12(3), 99–105. 10.1016/j.tics.2008.01.001, 18262825 PMC3632449

[bib68] Draganski, B., Kherif, F., Klöppel, S., Cook, P. A., Alexander, D. C., Parker, G. J. M., Deichmann, R., Ashburner, J., & Frackowiak, R. S. J. (2008). Evidence for segregated and integrative connectivity patterns in the human Basal Ganglia. Journal of Neuroscience, 28(28), 7143–7152. 10.1523/JNEUROSCI.1486-08.2008, 18614684 PMC6670486

[bib69] Drakesmith, M., Caeyenberghs, K., Dutt, A., Lewis, G., David, A. S., & Jones, D. K. (2015). Overcoming the effects of false positives and threshold bias in graph theoretical analyses of neuroimaging data. NeuroImage, 118, 313–333. 10.1016/j.neuroimage.2015.05.011, 25982515 PMC4558463

[bib70] Duncan, J. (2010). The multiple-demand (MD) system of the primate brain: mental programs for intelligent behaviour. Trends in Cognitive Sciences, 14(4), 172–179. 10.1016/j.tics.2010.01.004, 20171926

[bib71] Du, Y., & Fan, Y. (2013). Group information guided ICA for fMRI data analysis. NeuroImage, 69, 157–197. 10.1016/j.neuroimage.2012.11.008, 23194820

[bib72] Dworetsky, A., Seitzman, B. A., Adeyemo, B., Neta, M., Coalson, R. S., Petersen, S. E., & Gratton, C. (2021). Probabilistic mapping of human functional brain networks identifies regions of high group consensus. NeuroImage, 237, 118164. 10.1016/j.neuroimage.2021.118164, 34000397 PMC8296467

[bib73] Dworetsky, A., Seitzman, B. A., Adeyemo, B., Nielsen, A. N., Hatoum, A. S., Smith, D. M., Nichols, T. E., Neta, M., Petersen, S. E., & Gratton, C. (2023). Two common and distinct forms of variation in human functional brain networks. bioRxiv. 10.1101/2021.09.17.460799 PMC11248096 38689142

[bib74] Elliott, M. L., Knodt, A. R., Cooke, M., Kim, M. J., Melzer, T. R., Keenan, R., Ireland, D., Ramrakha, S., Poulton, R., Caspi, A., Moffitt, T. E., & Hariri, A. R. (2019). General functional connectivity: Shared features of resting-state and task fMRI drive reliable and heritable individual differences in functional brain networks. NeuroImage, 189, 516–532. 10.1016/j.neuroimage.2019.01.068, 30708106 PMC6462481

[bib75] Erhardt, E. B., Allen, E. A., Damaraju, E., & Calhoun, V. D. (2011). On network derivation, classification, and visualization: A response to Habeck and Moeller. Brain Connectivity, 1(2), 105–110. 10.1089/brain.2011.0022, 21808745 PMC3146759

[bib76] Esfahlani, F. Z., Jo, Y., Faskowitz, J., Byrge, L., Kennedy, D. P., Sporns, O., & Betzel, R. F. (2020). High-amplitude cofluctuations in cortical activity drive functional connectivity. Proceedings of the National Academy of Sciences of the United States of America, 117(45), 28393–28401. 10.1073/pnas.2005531117, 33093200 PMC7668041

[bib77] Faber, S. P., Timme, N. M., Beggs, J. M., & Newman, E. L. (2019). Computation is concentrated in rich clubs of local cortical networks. Network Neuroscience, 3(2), 384–404. 10.1162/netn_a_00069, 30793088 PMC6370472

[bib78] Fang, L., Zhang, L., Nie, D., Cao, X., Rekik, I., Lee, S.-W., He, H., & Shen, D. (2019). Automatic brain labeling via multi-atlas guided fully convolutional networks. Medical Image Analysis, 51, 157–168. 10.1016/j.media.2018.10.012, 30447544

[bib79] Faskowitz, J., Esfahlani, F. Z., Jo, Y., Sporns, O., & Betzel, R. F. (2020). Edge-centric functional network representations of human cerebral cortex reveal overlapping system-level architecture. Nature Neuroscience, 23(12), 1644–1654. 10.1038/s41593-020-00719-y, 33077948

[bib80] Favaretto, C., Spadone, S., Sestieri, C., Betti, V., Cenedese, A., Della Penna, S., & Corbetta, M. (2021). Multi-band MEG signatures of BOLD connectivity reorganization during visuospatial attention. NeuroImage, 230, 117781. 10.1016/j.neuroimage.2021.117781, 33497772

[bib81] Fedorenko, E. (2021). The early origins and the growing popularity of the individual-subject analytic approach in human neuroscience. Current Opinion in Behavioral Sciences, 40, 105–112. 10.1016/j.cobeha.2021.02.023

[bib82] Fedorenko, E., Duncan, J., & Kanwisher, N. (2013). Broad domain generality in focal regions of frontal and parietal cortex. Proceedings of the National Academy of Sciences of the United States of America, 110(41), 16616–16621. 10.1073/pnas.1315235110, 24062451 PMC3799302

[bib83] Feilong, M., Guntupalli, J. S., & Haxby, J. V. (2021). The neural basis of intelligence in fine-grained cortical topographies. eLife, 10, e64058. 10.7554/eLife.64058, 33683205 PMC7993992

[bib84] Felleman, D. J., & Van Essen, D. C. (1991). Distributed hierarchical processing in the primate cerebral cortex. Cerebral Cortex, 1(1), 1–47. 10.1093/cercor/1.1.1-a, 1822724

[bib85] Fornito, A., Harrison, B. J., Zalesky, A., & Simons, J. S. (2012). Competitive and cooperative dynamics of large-scale brain functional networks supporting recollection. Proceedings of the National Academy of Sciences of the United States of America, 109(31), 12788–12793. 10.1073/pnas.1204185109, 22807481 PMC3412011

[bib86] Fornito, A., Zalesky, A., & Bullmore, E. (2016). Fundamentals of brain network analysis. Academic Press.

[bib87] Friston, K. J. (1994). Functional and effective connectivity in neuroimaging: A synthesis. Human Brain Mapping, 2(1–2), 56–78. 10.1002/hbm.460020107

[bib88] Fritz, H.-C. J., Ray, N., Dyrba, M., Sorg, C., Teipel, S., & Grothe, M. J. (2019). The corticotopic organization of the human basal forebrain as revealed by regionally selective functional connectivity profiles. Human Brain Mapping, 40(3), 868–878. 10.1002/hbm.24417, 30311315 PMC6865372

[bib89] Fukushima, M., Betzel, R. F., He, Y., van den Heuvel, M. P., Zuo, X.-N., & Sporns, O. (2018). Structure-function relationships during segregated and integrated network states of human brain functional connectivity. Brain Structure & Function, 223(3), 1091–1106. 10.1007/s00429-017-1539-3, 29090337 PMC5871577

[bib90] Gilmore, A. W., Nelson, S. M., & McDermott, K. B. (2021). Precision functional mapping of human memory systems. Current Opinion in Behavioral Sciences, 40, 52–57. 10.1016/j.cobeha.2020.12.013

[bib91] Girard, G., Caminiti, R., Battaglia-Mayer, A., St-Onge, E., Ambrosen, K. S., Eskildsen, S. F., Krug, K., Dyrby, T. B., Descoteaux, M., Thiran, J.-P., & Innocenti, G. M. (2020). On the cortical connectivity in the macaque brain: A comparison of diffusion tractography and histological tracing data. NeuroImage, 221, 117201. 10.1016/j.neuroimage.2020.117201, 32739552

[bib92] Glasser, M. F., Coalson, T. S., Bijsterbosch, J. D., Harrison, S. J., Harms, M. P., Anticevic, A., Van Essen, D. C., & Smith, S. M. (2018). Using temporal ICA to selectively remove global noise while preserving global signal in functional MRI data. NeuroImage, 181, 692–717. 10.1016/j.neuroimage.2018.04.076, 29753843 PMC6237431

[bib93] Glasser, M. F., Coalson, T. S., Robinson, E. C., Hacker, C. D., Harwell, J., Yacoub, E., Ugurbil, K., Andersson, J., Beckmann, C. F., Jenkinson, M., Smith, S. M., & Van Essen, D. C. (2016). A multi-modal parcellation of human cerebral cortex. Nature, 536(7615), 171–178. 10.1038/nature18933, 27437579 PMC4990127

[bib94] Goñi, J., van den Heuvel, M. P., Avena-Koenigsberger, A., Velez de Mendizabal, N., Betzel, R. F., Griffa, A., Hagmann, P., Corominas-Murtra, B., Thiran, J.-P., & Sporns, O. (2014). Resting-brain functional connectivity predicted by analytic measures of network communication. Proceedings of the National Academy of Sciences of the United States of America, 111(2), 833–838. 10.1073/pnas.1315529111, 24379387 PMC3896172

[bib552] Gonzalez-Castillo, J., & Bandettini, P. A. (2018). Task-based dynamic functional connectivity: Recent findings and open questions. NeuroImage, 180(Pt B), 526–533. 10.1016/j.neuroimage.2017.08.006, 28780401 PMC5797523

[bib95] Gordon, E. M., Laumann, T. O., Adeyemo, B., Gilmore, A. W., Nelson, S. M., Dosenbach, N. U. F., & Petersen, S. E. (2017a). Individual-specific features of brain systems identified with resting state functional correlations. NeuroImage, 146, 918–939. 10.1016/j.neuroimage.2016.08.032, 27640749 PMC5321842

[bib96] Gordon, E. M., Laumann, T. O., Adeyemo, B., Huckins, J. F., Kelley, W. M., & Petersen, S. E. (2016). Generation and evaluation of a cortical area parcellation from resting-state correlations. Cerebral Cortex, 26(1), 288–303. 10.1093/cercor/bhu239, 25316338 PMC4677978

[bib97] Gordon, E. M., Laumann, T. O., Adeyemo, B., & Petersen, S. E. (2017b). Individual variability of the system-level organization of the human brain. Cerebral Cortex, 27(1), 386–399. 10.1093/cercor/bhv239, 26464473 PMC5939195

[bib98] Gordon, E. M., Laumann, T. O., Gilmore, A. W., Newbold, D. J., Greene, D. J., Berg, J. J., Ortega, M., Hoyt-Drazen, C., Gratton, C., Sun, H., Hampton, J. M., Coalson, R. S., Nguyen, A. L., McDermott, K. B., Shimony, J. S., Snyder, A. Z., Schlaggar, B. L., Petersen, S. E., Nelson, S. M., & Dosenbach, N. U. F. (2017c). Precision functional mapping of individual human brains. Neuron, 95(4), 791–807. 10.1016/j.neuron.2017.07.011, 28757305 PMC5576360

[bib99] Gordon, E. M., Laumann, T. O., Marek, S., Newbold, D. J., Hampton, J. M., Seider, N. A., Montez, D. F., Nielsen, A. M., Van, A. N., Zheng, A., Miller, R., Siegel, J. S., Kay, B. P., Snyder, A. Z., Greene, D. J., Schlaggar, B. L., Petersen, S. E., Nelson, S. M., & Dosenbach, N. U. F. (2022). Individualized functional subnetworks connect human striatum and frontal cortex. Cerebral Cortex, 32(13), 2868–2884. 10.1093/cercor/bhab387, 34718460 PMC9247416

[bib100] Gordon, E. M., Laumann, T. O., Marek, S., Raut, R. V., Gratton, C., Newbold, D. J., Greene, D. J., Coalson, R. S., Snyder, A. Z., Schlaggar, B. L., Petersen, S. E., Dosenbach, N. U. F., & Nelson, S. M. (2020). Default-mode network streams for coupling to language and control systems. Proceedings of the National Academy of Sciences of the United States of America, 117(29), 17308–17319. 10.1073/pnas.2005238117, 32632019 PMC7382234

[bib101] Gordon, E. M., & Nelson, S. M. (2021). Three types of individual variation in brain networks revealed by single-subject functional connectivity analyses. Current Opinion in Behavioral Sciences, 40, 79–86. 10.1016/j.cobeha.2021.02.014

[bib102] Gratton, C., Laumann, T. O., Gordon, E. M., Adeyemo, B., & Petersen, S. E. (2016). Evidence for two independent factors that modify brain networks to meet task goals. Cell Reports, 17(5), 1276–1288. 10.1016/j.celrep.2016.10.002, 27783943 PMC5123792

[bib103] Gratton, C., Laumann, T. O., Nielsen, A. N., Greene, D. J., Gordon, E. M., Gilmore, A. W., Nelson, S. M., Coalson, R. S., Snyder, A. Z., Schlaggar, B. L., Dosenbach, N. U. F., & Petersen, S. E. (2018). Functional brain networks are dominated by stable group and individual factors, not cognitive or daily variation. Neuron, 98(2), 439–452. 10.1016/j.neuron.2018.03.035, 29673485 PMC5912345

[bib104] Greene, D. J., Laumann, T. O., Dubis, J. W., Ihnen, S. K., Neta, M., Power, J. D., Pruett, J. R., Jr., Black, K. J., & Schlaggar, B. L. (2014). Developmental changes in the organization of functional connections between the basal ganglia and cerebral cortex. Journal of Neuroscience, 34(17), 5842–5854. 10.1523/JNEUROSCI.3069-13.2014, 24760844 PMC3996213

[bib105] Greene, D. J., Marek, S., Gordon, E. M., Siegel, J. S., Gratton, C., Laumann, T. O., Gilmore, A. W., Berg, J. J., Nguyen, A. L., Dierker, D., Van, A. N., Ortega, M., Newbold, D. J., Hampton, J. M., Nielsen, A. N., McDermott, K. B., Roland, J. L., Norris, S. A., Nelson, S. M., … Dosenbach, N. U. F. (2020). Integrative and network-specific connectivity of the basal ganglia and thalamus defined in individuals. Neuron, 105(4), 742–758. 10.1016/j.neuron.2019.11.012, 31836321 PMC7035165

[bib106] Greicius, M. D., Krasnow, B., Reiss, A. L., & Menon, V. (2003). Functional connectivity in the resting brain: a network analysis of the default mode hypothesis. Proceedings of the National Academy of Sciences of the United States of America, 100(1), 253–258. 10.1073/pnas.0135058100, 12506194 PMC140943

[bib107] Greicius, M. D., Srivastava, G., Reiss, A. L., & Menon, V. (2004). Default-mode network activity distinguishes Alzheimer’s disease from healthy aging: Evidence from functional MRI. Proceedings of the National Academy of Sciences of the United States of America, 101(13), 4637–4642. 10.1073/pnas.0308627101, 15070770 PMC384799

[bib108] Grisot, G., Haber, S. N., & Yendiki, A. (2021). Diffusion MRI and anatomic tracing in the same brain reveal common failure modes of tractography. NeuroImage, 239, 118300. 10.1016/j.neuroimage.2021.118300, 34171498 PMC8475636

[bib109] Gu, S., Satterthwaite, T. D., Medaglia, J. D., Yang, M., Gur, R. E., Gur, R. C., & Bassett, D. S. (2015). Emergence of system roles in normative neurodevelopment. Proceedings of the National Academy of Sciences of the United States of America, 112(44), 13681–13686. 10.1073/pnas.1502829112, 26483477 PMC4640772

[bib110] Haak, K. V., & Beckmann, C. F. (2020). Understanding brain organisation in the face of functional heterogeneity and functional multiplicity. NeuroImage, 220, 117061. 10.1016/j.neuroimage.2020.117061, 32574808

[bib111] Haber, S. N. (2010). Integrative networks across basal ganglia circuits. In H. Steiner & K. Y. Tseng (Eds.), Handbook of behavioral neuroscience (Vol. 20, pp. 409–427). Elsevier. 10.1016/B978-0-12-374767-9.00024-X

[bib112] Hagmann, P., Cammoun, L., Gigandet, X., Meuli, R., Honey, C. J., Wedeen, V. J., & Sporns, O. (2008). Mapping the structural core of human cerebral cortex. PLoS Biology, 6(7), e159. 10.1371/journal.pbio.0060159, 18597554 PMC2443193

[bib113] Han, L., Savalia, N. K., Chan, M. Y., Agres, P. F., Nair, A. S., & Wig, G. S. (2018). Functional parcellation of the cerebral cortex across the human adult lifespan. Cerebral Cortex, 28(12), 4403–4423. 10.1093/cercor/bhy218, 30307480 PMC6215466

[bib114] Hari, R., & Parkkonen, L. (2015). The brain timewise: How timing shapes and supports brain function. Philosophical Transactions of the Royal Society of London Series B: Biological Sciences, 370(1668), 20140170. 10.1098/rstb.2014.0170, 25823867 PMC4387511

[bib115] Harrison, S. J., Woolrich, M. W., Robinson, E. C., Glasser, M. F., Beckmann, C. F., Jenkinson, M., & Smith, S. M. (2015). Large-scale probabilistic functional modes from resting state fMRI. NeuroImage, 109, 217–231. 10.1016/j.neuroimage.2015.01.013, 25598050 PMC4349633

[bib116] Hayashi, T., Hou, Y., Glasser, M. F., Autio, J. A., Knoblauch, K., Inoue-Murayama, M., Coalson, T., Yacoub, E., Smith, S., Kennedy, H., & Van Essen, D. C. (2021). The nonhuman primate neuroimaging and neuroanatomy project. NeuroImage, 229, 117726. 10.1016/j.neuroimage.2021.117726, 33484849 PMC8079967

[bib117] Hearne, L. J., Cocchi, L., Zalesky, A., & Mattingley, J. B. (2017). Reconfiguration of brain network architectures between resting-state and complexity-dependent cognitive reasoning. Journal of Neuroscience, 37(35), 8399–8411. 10.1523/JNEUROSCI.0485-17.2017, 28760864 PMC6596866

[bib118] He, B. J., Snyder, A. Z., Zempel, J. M., Smyth, M. D., & Raichle, M. E. (2008). Electrophysiological correlates of the brain’s intrinsic large-scale functional architecture. Proceedings of the National Academy of Sciences of the United States of America, 105(41), 16039–16044. 10.1073/pnas.0807010105, 18843113 PMC2564983

[bib119] Hermosillo, R. J. M., Moore, L. A., Fezcko, E., Dworetsky, A., Pines, A., Conan, G., Mooney, M. A., Randolph, A., Adeyemo, B., Earl, E., Perrone, A., Carrasco, C. M., Uriarte-Lopez, J., Snider, K., Doyle, O., Cordova, M., Nagel, B. J., Feldstein Ewing, S. W., Satterthwaite, T., … Fair, D. A. (2022). A precision functional atlas of network probabilities and individual-specific network topography. bioRxiv. 10.1101/2022.01.12.475422

[bib120] Hiser, J., & Koenigs, M. (2018). The multifaceted role of the ventromedial prefrontal cortex in emotion, decision making, social cognition, and psychopathology. Biological Psychiatry, 83(8), 638–647. 10.1016/j.biopsych.2017.10.030, 29275839 PMC5862740

[bib121] Honey, C. J., Kötter, R., Breakspear, M., & Sporns, O. (2007). Network structure of cerebral cortex shapes functional connectivity on multiple time scales. Proceedings of the National Academy of Sciences of the United States of America, 104(24), 10240–10245. 10.1073/pnas.0701519104, 17548818 PMC1891224

[bib122] Honey, C. J., Sporns, O., Cammoun, L., Gigandet, X., Thiran, J. P., Meuli, R., & Hagmann, P. (2009). Predicting human resting-state functional connectivity from structural connectivity. Proceedings of the National Academy of Sciences of the United States of America, 106(6), 2035–2040. 10.1073/pnas.0811168106, 19188601 PMC2634800

[bib123] Honey, C. J., Thivierge, J.-P., & Sporns, O. (2010). Can structure predict function in the human brain? NeuroImage, 52(3), 766–776. 10.1016/j.neuroimage.2010.01.071, 20116438

[bib124] Hugdahl, K., Raichle, M. E., Mitra, A., & Specht, K. (2015). On the existence of a generalized non-specific task-dependent network. Frontiers in Human Neuroscience, 9, 430. 10.3389/fnhum.2015.00430, 26300757 PMC4526816

[bib125] Hu, G., Waters, A. B., Aslan, S., Frederick, B., Cong, F., & Nickerson, L. D. (2020). Snowball ICA: A model order free independent component analysis strategy for functional magnetic resonance imaging data. Frontiers in Neuroscience, 14, 569657. 10.3389/fnins.2020.569657, 33071741 PMC7530342

[bib126] Huntenburg, J. M., Bazin, P.-L., & Margulies, D. S. (2018). Large-scale gradients in human cortical organization. Trends in Cognitive Sciences, 22(1), 21–31. 10.1016/j.tics.2017.11.002, 29203085

[bib127] Hutchison, R. M., Womelsdorf, T., Allen, E. A., Bandettini, P. A., Calhoun, V. D., Corbetta, M., Della Penna, S., Duyn, J. H., Glover, G. H., Gonzalez-Castillo, J., Handwerker, D. A., Keilholz, S., Kiviniemi, V., Leopold, D. A., de Pasquale, F., Sporns, O., Walter, M., & Chang, C. (2013). Dynamic functional connectivity: Promise, issues, and interpretations. NeuroImage, 80, 360–378. 10.1016/j.neuroimage.2013.05.079, 23707587 PMC3807588

[bib128] Iraji, A., Faghiri, A., Fu, Z., Kochunov, P., Adhikari, B. M., Belger, A., Ford, J. M, McEwen, S., Mathalon, D. H., Pearlson, G. D., Potkin, S. G., Preda, A., Turner, J. A., Van Erp, T. G. M., Chang, C., & Calhoun, V. D. (2022). Moving beyond the ‘CAP’ of the Iceberg: Intrinsic connectivity networks in fMRI are continuously engaging and overlapping. NeuroImage, 251, 119013. 10.1016/j.neuroimage.2022.119013, 35189361 PMC9107614

[bib129] Ivanova, A., Zaidel, E., Salamon, N., Bookheimer, S., Uddin, L. Q., & de Bode, S. (2017). Intrinsic functional organization of putative language networks in the brain following left cerebral hemispherectomy. Brain Structure & Function, 222(8), 3795–3805. 10.1007/s00429-017-1434-y, 28470553 PMC6032986

[bib130] Jarbo, K., & Verstynen, T. D. (2015). Converging structural and functional connectivity of orbitofrontal, dorsolateral prefrontal, and posterior parietal cortex in the human striatum. Journal of Neuroscience, 35(9), 3865–3878. 10.1523/JNEUROSCI.2636-14.2015, 25740516 PMC4461697

[bib131] Jo, Y., Zamani Esfahlani, F., Faskowitz, J., Chumin, E. J., Sporns, O., & Betzel, R. F. (2021). The diversity and multiplexity of edge communities within and between brain systems. Cell Reports, 37(7), 110032. 10.1016/j.celrep.2021.110032, 34788617

[bib132] Jung, K., Eickhoff, S. B., & Popovych, O. V. (2021). Tractography density affects whole-brain structural architecture and resting-state dynamical modeling. NeuroImage, 237, 118176. 10.1016/j.neuroimage.2021.118176, 34000399

[bib133] Kantarovich, K., Mwilambwe-Tshilobo, L., Fernández-Cabello, S., Setton, R., Baracchini, G., Lockrow, A. W., Spreng, R. N., & Turner, G. R. (2022). White matter lesion load is associated with lower within- and greater between-network connectivity across older age. Neurobiology of Aging, 112, 170–180. 10.1016/j.neurobiolaging.2022.01.005, 35219126

[bib134] Kanwisher, N., McDermott, J., & Chun, M. M. (1997). The fusiform face area: A module in human extrastriate cortex specialized for face perception. Journal of Neuroscience, 17(11), 4302–4311. 10.1523/JNEUROSCI.17-11-04302.1997, 9151747 PMC6573547

[bib135] King, M., Hernandez-Castillo, C. R., Poldrack, R. A., Ivry, R. B., & Diedrichsen, J. (2019). Functional boundaries in the human cerebellum revealed by a multi-domain task battery. Nature Neuroscience, 22(8), 1371–1378. 10.1038/s41593-019-0436-x, 31285616 PMC8312478

[bib553] Kinnison, J., Padmala, S., Choi, J. M., & Pessoa, L. (2012). Network analysis reveals increased integration during emotional and motivational processing. Journal of Neuroscience, 32(24), 8361–8372. 10.1523/JNEUROSCI.0821-12.2012, 22699916 PMC3400262

[bib136] Kiviniemi, V., Kantola, J.-H., Jauhiainen, J., Hyvärinen, A., & Tervonen, O. (2003). Independent component analysis of nondeterministic fMRI signal sources. NeuroImage, 19(2), 253–260. 10.1016/S1053-8119(03)00097-1, 12814576

[bib137] Kiviniemi, V., Starck, T., Remes, J., Long, X., Nikkinen, J., Haapea, M., Veijola, J., Moilanen, I., Isohanni, M., Zang, Y.-F., & Tervonen, O. (2009). Functional segmentation of the brain cortex using high model order group PICA. Human Brain Mapping, 30(12), 3865–3886. 10.1002/hbm.20813, 19507160 PMC6870574

[bib138] Kliemann, D., Adolphs, R., Tyszka, J. M., Fischl, B., Yeo, B. T. T., Nair, R., Dubois, J., & Paul, L. K. (2019). Intrinsic functional connectivity of the brain in adults with a single cerebral hemisphere. Cell Reports, 29(8), 2398–2407. 10.1016/j.celrep.2019.10.067, 31747608 PMC6914265

[bib139] Kong, R., Li, J., Orban, C., Sabuncu, M. R., Liu, H., Schaefer, A., Sun, N., Zuo, X.-N., Holmes, A. J., Eickhoff, S. B., & Yeo, B. T. T. (2019). Spatial topography of individual-specific cortical networks predicts human cognition, personality, and emotion. Cerebral Cortex, 29(6), 2533–2551. 10.1093/cercor/bhy123, 29878084 PMC6519695

[bib140] Kong, R., Spreng, R. N., Nickerson, L., Fornito, A., Laird, A., Razi, A., Yendiki, A., Gratton, C., Gordon, E., Larson-Prior, L., Cohen, J., Damoiseaux, J., Betzel, R., Eickhoff, S., Sadaghiani, S., Uddin, L. Q., & Yeo, B. T. T. (2022). Correspondences across 16 group-level functional brain network atlases [Paper presentation]. Annual Meeting of Organization for Human Brain Mapping.

[bib141] Kong, R., Yang, Q., Gordon, E., Xue, A., Yan, X., Orban, C., Zuo, X.-N., Spreng, N., Ge, T., Holmes, A., Eickhoff, S., & Yeo, B. T. T. (2021). Individual-specific areal-level parcellations improve functional connectivity prediction of behavior. Cerebral Cortex, 31(10), 4477–4500. 10.1093/cercor/bhab101, 33942058 PMC8757323

[bib142] Kraus, B. T., Perez, D., Ladwig, Z., Seitzman, B. A., Dworetsky, A., Petersen, S. E., & Gratton, C. (2021). Network variants are similar between task and rest states. NeuroImage, 229, 117743. 10.1016/j.neuroimage.2021.117743, 33454409 PMC8080895

[bib143] Krienen, F. M., Yeo, B. T. T., & Buckner, R. L. (2014). Reconfigurable task-dependent functional coupling modes cluster around a core functional architecture. Philosophical Transactions of the Royal Society of London Series B: Biological Sciences, 369(1653), 20130526. 10.1098/rstb.2013.0526, 25180304 PMC4150301

[bib144] Kucyi, A., Schrouff, J., Bickel, S., Foster, B. L., Shine, J. M., & Parvizi, J. (2018). Intracranial electrophysiology reveals reproducible intrinsic functional connectivity within human brain networks. Journal of Neuroscience, 38(17), 4230–4242. 10.1523/JNEUROSCI.0217-18.2018, 29626167 PMC5963853

[bib145] Kundu, P., Voon, V., Balchandani, P., Lombardo, M. V., Poser, B. A., & Bandettini, P. A. (2017). Multi-echo fMRI: A review of applications in fMRI denoising and analysis of BOLD signals. NeuroImage, 154, 59–80. 10.1016/j.neuroimage.2017.03.033, 28363836

[bib146] Ladwig, Z., Seitzman, B. A., Dworetsky, A., Yu, Y., Adeyemo, B., Smith, D. M., Petersen, S. E., & Gratton, C. (2022). BOLD cofluctuation “events” are predicted from static functional connectivity. NeuroImage, 260, 119476. 10.1016/j.neuroimage.2022.119476, 35842100 PMC9428936

[bib147] Lashley, K. S., & Clark, G. (1946). The cytoarchitecture of the cerebral cortex of Ateles; A critical examination of architectonic studies. Journal of Comparative Neurology, 85(2), 223–305. 10.1002/cne.900850207, 21002789

[bib148] Laumann, T. O., Gordon, E. M., Adeyemo, B., Snyder, A. Z., Joo, S. J., Chen, M.-Y., Gilmore, A. W., McDermott, K. B., Nelson, S. M., Dosenbach, N. U. F., Schlaggar, B. L., Mumford, J. A., Poldrack, R. A., & Petersen, S. E. (2015). Functional system and areal organization of a highly sampled individual human brain. Neuron, 87(3), 657–670. 10.1016/j.neuron.2015.06.037, 26212711 PMC4642864

[bib149] Laumann, T. O., Ortega, M., Hoyt, C. R., Seider, N. A., Snyder, A. Z., Dosenbach, N. U. F., & Brain Network Plasticity Group. (2021). Brain network reorganisation in an adolescent after bilateral perinatal strokes. Lancet Neurology, 20(4), 255–256. 10.1016/S1474-4422(21)00062-4, 33743230 PMC13182026

[bib150] Laumann, T. O., Snyder, A. Z., Mitra, A., Gordon, E. M., Gratton, C., Adeyemo, B., Gilmore, A. W., Nelson, S. M., Berg, J. J., Greene, D. J., McCarthy, J. E., Tagliazucchi, E., Laufs, H., Schlaggar, B. L., Dosenbach, N. U. F., & Petersen, S. E. (2017). On the stability of BOLD fMRI correlations. Cerebral Cortex, 27(10), 4719–4732. 10.1093/cercor/bhw265, 27591147 PMC6248456

[bib151] Lee, J.-H., Lee, T.-W., Jolesz, F. A., & Yoo, S.-S. (2008). Independent vector analysis (IVA): Multivariate approach for fMRI group study. NeuroImage, 40(1), 86–109. 10.1016/j.neuroimage.2007.11.019, 18165105

[bib152] Li, J., Curley, W. H., Guerin, B., Dougherty, D. D., Dalca, A. V., Fischl, B., Horn, A., & Edlow, B. L. (2021). Mapping the subcortical connectivity of the human default mode network. NeuroImage, 245, 118758. 10.1016/j.neuroimage.2021.118758, 34838949 PMC8945548

[bib153] Liu, Z.-Q., Rodriguez-Vazquez, B., Spreng, R. N., Bernhardt, B. C., Betzel, R. F. & Misic, B. (2022). Time-resolved structure-function coupling in brain networks. Communications Biology, 5(1), 532. 10.1038/s42003-022-03466-x, 35654886 PMC9163085

[bib154] Lu, H., Zou, Q., Gu, H., Raichle, M. E., Stein, E. A., & Yang, Y. (2012). Rat brains also have a default mode network. Proceedings of the National Academy of Sciences of the United States of America, 109(10), 3979–3984. 10.1073/pnas.1200506109, 22355129 PMC3309754

[bib155] Luca, M. D., Beckmann, C. F., De Stefano, N., Matthews, P. M., & Smith, S. M. (2006). fMRI resting state networks define distinct modes of long-distance interactions in the human brain. NeuroImage, 29(4), 1359–1367. 10.1016/j.neuroimage.2005.08.035, 16260155

[bib156] Lurie, D. J., Kessler, D., Bassett, D. S., Betzel, R. F., Breakspear, M., Kheilholz, S., Kucyi, A., Liégeois, R., Lindquist, M. A., McIntosh, A. R., Poldrack, R. A., Shine, J. M., Thompson, W. H., Bielczyk, N. Z., Douw, L., Kraft, D., Miller, R. L., Muthuraman, M., Pasquini, L., … Calhoun, V. D. (2020). Questions and controversies in the study of time-varying functional connectivity in resting fMRI. Network Neuroscience, 4(1), 30–69. 10.1162/netn_a_00116, 32043043 PMC7006871

[bib157] Lynch, C. J., Power, J. D., Scult, M. A., Dubin, M., Gunning, F. M., & Liston, C. (2020a). Rapid precision functional mapping of individuals using multi-echo fMRI. Cell Reports, 33(12), 108540. 10.1016/j.celrep.2020.108540, 33357444 PMC7792478

[bib158] Lynch, C. J., Silver, B. M., Dubin, M. J., Martin, A., Voss, H. U., Jones, R. M., & Power, J. D. (2020b). Prevalent and sex-biased breathing patterns modify functional connectivity MRI in young adults. Nature Communications, 11(1), 5290. 10.1038/s41467-020-18974-9, 33082311 PMC7576607

[bib159] Maffei, C., Girard, G., Schilling, K. G., Aydogan, D. B., Adluru, N., Zhylka, A., Wu, Y., Mancini, M., Hamamci, A., Sarica, A., Teillac, A., Baete, S. H., Karimi, D., Yeh, F.-C., Yildiz, M. E., Gholipour, A., Bihan-Poudec, Y., Hiba, B., Quattrone, A., … Yendiki, A. (2022). Insights from the IronTract challenge: Optimal methods for mapping brain pathways from multi-shell diffusion MRI. NeuroImage, 257, 119327. 10.1016/j.neuroimage.2022.119327, 35636227 PMC9453851

[bib160] Maier-Hein, K. H., Neher, P. F., Houde, J.-C., Côté, M.-A., Garyfallidis, E., Zhong, J., Chamberland, M., Yeh, F.-C., Lin, Y.-C., Ji, Q., Reddick, W. E., Glass, J. O., Chen, D. Q., Feng, Y., Gao, C., Wu, Y., Ma, J., He, R., Li, Q., … Descoteaux, M. (2017). The challenge of mapping the human connectome based on diffusion tractography. Nature Communications, 8(1), 1349. 10.1038/s41467-017-01285-x, 29116093 PMC5677006

[bib161] Mantini, D., Gerits, A., Nelissen, K., Durand, J.-B., Joly, O., Simone, L., Sawamura, H., Wardak, C., Orban, G. A., Buckner, R. L., & Vanduffel, W. (2011). Default mode of brain function in monkeys. Journal of Neuroscience, 31(36), 12954–12962. 10.1523/JNEUROSCI.2318-11.2011, 21900574 PMC3686636

[bib162] Marek, S., Siegel, J. S., Gordon, E. M., Raut, R. V., Gratton, C., Newbold, D. J., Ortega, M., Laumann, T. O., Adeyemo, B., Miller, D. B., Zheng, A., Lopez, K. C., Berg, J. J., Coalson, R. S., Nguyen, A. L., Dierker, D., Van, A. N., Hoyt, C. R., McDermott, K. B., … Dosenbach, N. U. F. (2018). Spatial and temporal organization of the individual human cerebellum. Neuron, 100(4), 977–993. 10.1016/j.neuron.2018.10.010, 30473014 PMC6351081

[bib163] Marek, S., Tervo-Clemmens, B., Calabro, F. J., Montez, D. F., Kay, B. P., Hatoum, A. S., Donohue, M. R., Foran, W., Miller, R. L., Feczko, E., Miranda-Dominguez, O., Graham, A. M., Earl, E. A., Perrone, A. J., Cordova, M., Doyle, O., Moore, L. A., Conan, G., Uriarte, J., … Dosenbach, N. U. F. (2020). Towards reproducible brain-wide association studies. bioRxiv. 10.1101/2020.08.21.257758

[bib164] Markello, R. D., Spreng, R. N., Luh, W.-M., Anderson, A. K., & De Rosa, E. (2018). Segregation of the human basal forebrain using resting state functional MRI. NeuroImage, 173, 287–297. 10.1016/j.neuroimage.2018.02.042, 29496614

[bib554] McMenamin, B. W., Langeslag, S. J., Sirbu, M., Padmala, S., & Pessoa, L. (2014). Network organization unfolds over time during periods of anxious anticipation. Journal of Neuroscience, 34(34), 11261–11273. 10.1523/JNEUROSCI.1579-14.2014, 25143607 PMC4138337

[bib165] Mejia, A. F., Nebel, M. B., Wang, Y., Caffo, B. S., & Guo, Y. (2020). Template independent component analysis: Targeted and reliable estimation of subject-level brain networks using big data population priors. Journal of the American Statistical Association, 115(531), 1151–1177. 10.1080/01621459.2019.1679638, 33060872 PMC7556739

[bib166] Melloni, L., Mudrik, L., Pitts, M., & Koch, C. (2021). Making the hard problem of consciousness easier. Science, 372(6545), 911–912. 10.1126/science.abj3259, 34045342

[bib167] Messé, A., Rudrauf, D., Benali, H., & Marrelec, G. (2014). Relating structure and function in the human brain: Relative contributions of anatomy, stationary dynamics, and non-stationarities. PLoS Computational Biology, 10(3), e1003530. 10.1371/journal.pcbi.1003530, 24651524 PMC3961181

[bib168] Mesulam, M. M. (1998). From sensation to cognition. Brain, 121(6), 1013–1052. 10.1093/brain/121.6.1013, 9648540

[bib169] Metzak, P., Feredoes, E., Takane, Y., Wang, L., Weinstein, S., Cairo, T., Ngan, E. T. C., & Woodward, T. S. (2011). Constrained principal component analysis reveals functionally connected load-dependent networks involved in multiple stages of working memory. Human Brain Mapping, 32(6), 856–871. 10.1002/hbm.21072, 20572208 PMC6870062

[bib170] Michael, A. M., Anderson, M., Miller, R. L., Adalı, T., & Calhoun, V. D. (2014). Preserving subject variability in group fMRI analysis: Performance evaluation of GICA vs. IVA. Frontiers in Systems Neuroscience, 8, 106. 10.3389/fnsys.2014.00106, 25018704 PMC4071815

[bib171] Mountcastle, V. B. (1997). The columnar organization of the neocortex. Brain, 120(4), 701–722. 10.1093/brain/120.4.701, 9153131

[bib172] Mueller, S., Wang, D., Fox, M. D., Yeo, B. T. T., Sepulcre, J., Sabuncu, M. R., Shafee, R., Lu, J., & Liu, H. (2013). Individual variability in functional connectivity architecture of the human brain. Neuron, 77(3), 586–595. 10.1016/j.neuron.2012.12.028, 23395382 PMC3746075

[bib555] Najafi, M., Kinnison, J., & Pessoa, L. (2017). Dynamics of intersubject brain networks during anxious anticipation. Frontiers in Human Neuroscience, 11, 552. 10.3389/fnhum.2017.00552, 29209184 PMC5702479

[bib173] Najafi, M., McMenamin, B. W., Simon, J. Z., & Pessoa, L. (2016). Overlapping communities reveal rich structure in large-scale brain networks during rest and task conditions. NeuroImage, 135, 92–106. 10.1016/j.neuroimage.2016.04.054, 27129758 PMC4915991

[bib174] Nichols, T. E., Das, S., Eickhoff, S. B., Evans, A. C., Glatard, T., Hanke, M., Kriegeskorte, N., Milham, M. P., Poldrack, R. A., Poline, J.-B., Proal, E., Thirion, B., Van Essen, D. C., White, T., & Yeo, B. T. T. (2017). Best practices in data analysis and sharing in neuroimaging using MRI. Nature Neuroscience, 20(3), 299–303. 10.1038/nn.4500, 28230846 PMC5685169

[bib175] Nickerson, L. D., Smith, S. M., Öngür, D., & Beckmann, C. F. (2017). Using dual regression to investigate network shape and amplitude in functional connectivity analyses. Frontiers in Neuroscience, 11, 115. 10.3389/fnins.2017.00115, 28348512 PMC5346569

[bib176] Nielsen, A. N., Greene, D. J., Gratton, C., Dosenbach, N. U. F., Petersen, S. E., & Schlaggar, B. L. (2019). Evaluating the prediction of brain maturity from functional connectivity after motion artifact denoising. Cerebral Cortex, 29(6), 2455–2469. 10.1093/cercor/bhy117, 29850877 PMC6519700

[bib177] Niendam, T. A., Laird, A. R., Ray, K. L., Dean, Y. M., Glahn, D. C., & Carter, C. S. (2012). Meta-analytic evidence for a superordinate cognitive control network subserving diverse executive functions. Cognitive, Affective, & Behavioral Neuroscience, 12(2), 241–268. 10.3758/s13415-011-0083-5, 22282036 PMC3660731

[bib178] Nieuwenhuys, R. (2013). The myeloarchitectonic studies on the human cerebral cortex of the Vogt-Vogt school, and their significance for the interpretation of functional neuroimaging data. Brain Structure & Function, 218(2), 303–352. 10.1007/s00429-012-0460-z, 23076375

[bib179] Nir, Y., Mukamel, R., Dinstein, I., Privman, E., Harel, M., Fisch, L., Gelbard-Sagiv, H., Kipervasser, S., Andelman, F., Neufeld, M. Y., Kramer, U., Arieli, A., Fried, I., & Malach, R. (2008). Interhemispheric correlations of slow spontaneous neuronal fluctuations revealed in human sensory cortex. Nature Neuroscience, 11(9), 1100–1108. 10.1038/nn.2177, 19160509 PMC2642673

[bib180] Noble, S., Spann, M. N., Tokoglu, F., Shen, X., Constable, R. T., & Scheinost, D. (2017). Influences on the test–retest reliability of functional connectivity MRI and its relationship with behavioral utility. Cerebral Cortex, 27(11), 5415–5429. 10.1093/cercor/bhx230, 28968754 PMC6248395

[bib181] Nomi, J. S., Marshall, E., Zaidel, E., Biswal, B., Castellanos, F. X., Dick, A. S., Uddin, L. Q., & Mooshagian, E. (2019). Diffusion weighted imaging evidence of extra-callosal pathways for interhemispheric communication after complete commissurotomy. Brain Structure & Function, 224(5), 1897–1909. 10.1007/s00429-019-01864-2, 31062161 PMC6565442

[bib182] Oldham, S., Arnatkevic Iūtė, A., Smith, R. E., Tiego, J., Bellgrove, M. A., & Fornito, A. (2020). The efficacy of different preprocessing steps in reducing motion-related confounds in diffusion MRI connectomics. NeuroImage, 222, 117252. 10.1016/j.neuroimage.2020.117252, 32800991

[bib183] Orban, C., Kong, R., Li, J., Chee, M. W. L., & Yeo, B. T. T. (2020). Time of day is associated with paradoxical reductions in global signal fluctuation and functional connectivity. PLoS Biology, 18(2), e3000602. 10.1371/journal.pbio.3000602, 32069275 PMC7028250

[bib184] Palla, G., Derényi, I., Farkas, I., & Vicsek, T. (2005). Uncovering the overlapping community structure of complex networks in nature and society. Nature, 435(7043), 814–818. 10.1038/nature03607, 15944704

[bib185] Pandya, D. N., & Yeterian, E. H. (1985). Architecture and connections of cortical association areas. In A. Peters & E. G. Jones (Eds.), Association and auditory cortices (pp. 3–61). Springer. 10.1007/978-1-4757-9619-3_1

[bib186] Parker, C. S., Deligianni, F., Cardoso, M. J., Daga, P., Modat, M., Dayan, M., Clark, C. A., Ourselin, S., & Clayden, J. D. (2014). Consensus between pipelines in structural brain networks. PLoS One, 9(10), e111262. 10.1371/journal.pone.0111262, 25356977 PMC4214749

[bib187] Parkes, L., Fulcher, B., Yücel, M., & Fornito, A. (2018). An evaluation of the efficacy, reliability, and sensitivity of motion correction strategies for resting-state functional MRI. NeuroImage, 171, 415–436. 10.1016/j.neuroimage.2017.12.073, 29278773

[bib188] Pernet, C., Garrido, M. I., Gramfort, A., Maurits, N., Michel, C. M., Pang, E., Salmelin, R., Schoffelen, J. M., Valdes-Sosa, P. A., & Puce, A. (2020). Issues and recommendations from the OHBM COBIDAS MEEG committee for reproducible EEG and MEG research. Nature Neuroscience, 23(12), 1473–1483. 10.1038/s41593-020-00709-0, 32958924

[bib189] Pervaiz, U., Vidaurre, D., Woolrich, M. W., & Smith, S. M. (2020). Optimising network modelling methods for fMRI. NeuroImage, 211, 116604. 10.1016/j.neuroimage.2020.116604, 32062083 PMC7086233

[bib190] Pessoa, L. (2014). Understanding brain networks and brain organization. Physics of Life Reviews, 11(3), 400–435. 10.1016/j.plrev.2014.03.005, 24819881 PMC4157099

[bib191] Petrides, M., Tomaiuolo, F., Yeterian, E. H., & Pandya, D. N. (2012). The prefrontal cortex: Comparative architectonic organization in the human and the macaque monkey brains. Cortex, 48(1), 46–57. 10.1016/j.cortex.2011.07.002, 21872854

[bib192] Power, J. D., Barnes, K. A., Snyder, A. Z., Schlaggar, B. L., & Petersen, S. E. (2012). Spurious but systematic correlations in functional connectivity MRI networks arise from subject motion. NeuroImage, 59(3), 2142–2154. 10.1016/j.neuroimage.2011.10.018, 22019881 PMC3254728

[bib193] Power, J. D., Cohen, A. L., Nelson, S. M., Wig, G. S., Barnes, K. A., Church, J. A., Vogel, A. C., Laumann, T. O., Miezin, F. M., Schlaggar, B. L., & Petersen, S. E. (2011a). Functional network organization of the human brain. Neuron, 72(4), 665–678. 10.1016/j.neuron.2011.09.006, 22099467 PMC3222858

[bib194] Power, J. D., Fair, D. A., Schlaggar, B. L., & Petersen, S. E. (2011b). The development of human functional brain networks. Neuron, 67(5), 735–748. 10.1016/j.neuron.2010.08.017, 20826306 PMC2941973

[bib195] Power, J. D., Lynch, C. J., Adeyemo, B., & Petersen, S. E. (2020). A critical, event-related appraisal of denoising in resting-state fMRI studies. Cerebral Cortex, 30(10), 5544–5559. 10.1093/cercor/bhaa139, 32494823

[bib196] Power, J. D., Mitra, A., Laumann, T. O., Snyder, A. Z., Schlaggar, B. L., & Petersen, S. E. (2014). Methods to detect, characterize, and remove motion artifact in resting state fMRI. NeuroImage, 84, 320–341. 10.1016/j.neuroimage.2013.08.048, 23994314 PMC3849338

[bib197] Power, J. D., Plitt, M., Gotts, S. J., Kundu, P., Voon, V., Bandettini, P. A., & Martin, A. (2018). Ridding fMRI data of motion-related influences: Removal of signals with distinct spatial and physical bases in multiecho data. Proceedings of the National Academy of Sciences of the United States of America, 115(9), E2105–E2114. 10.1073/pnas.1720985115, 29440410 PMC5834724

[bib198] Power, J. D., Plitt, M., Laumann, T. O., & Martin, A. (2017). Sources and implications of whole-brain fMRI signals in humans. NeuroImage, 146, 609–625. 10.1016/j.neuroimage.2016.09.038, 27751941 PMC5321814

[bib199] Raichle, M. E., MacLeod, A. M., Snyder, A. Z., Powers, W. J., Gusnard, D. A., & Shulman, G. L. (2001). A default mode of brain function. Proceedings of the National Academy of Sciences of the United States of America, 98(2), 676–682. 10.1073/pnas.98.2.676, 11209064 PMC14647

[bib200] Ramnani, N. (2006). The primate cortico-cerebellar system: Anatomy and function. Nature Reviews Neuroscience, 7(7), 511–522. 10.1038/nrn1953, 16791141

[bib201] Reid, A. T., Headley, D. B., Mill, R. D., Sanchez-Romero, R., Uddin, L. Q., Marinazzo, D., Lurie, D. J., Valdés-Sosa, P. A., Hanson, S. J., Biswal, B. B., Calhoun, V., Poldrack, R. A., & Cole, M. W. (2019). Advancing functional connectivity research from association to causation. Nature Neuroscience, 22(11), 1751–1760. 10.1038/s41593-019-0510-4, 31611705 PMC7289187

[bib202] Rolls, E. T., Huang, C.-C., Lin, C.-P., Feng, J., & Joliot, M. (2020). Automated anatomical labelling atlas 3. NeuroImage, 206, 116189. 10.1016/j.neuroimage.2019.116189, 31521825

[bib203] Rosenthal, G., Váša, F., Griffa, A., Hagmann, P., Amico, E., Goñi, J., Avidan, G., & Sporns, O. (2018). Mapping higher-order relations between brain structure and function with embedded vector representations of connectomes. Nature Communications, 9(1), 2178. 10.1038/s41467-018-04614-w, 29872218 PMC5988787

[bib204] Rubinov, M., & Sporns, O. (2010). Complex network measures of brain connectivity: Uses and interpretations. NeuroImage, 52(3), 1059–1069. 10.1016/j.neuroimage.2009.10.003, 19819337

[bib205] Sadaghiani, S., & Wirsich, J. (2020). Intrinsic connectome organization across temporal scales: New insights from cross-modal approaches. Network Neuroscience, 4(1), 1–29. 10.1162/netn_a_00114, 32043042 PMC7006873

[bib206] Salehi, M., Greene, A. S., Karbasi, A., Shen, X., Scheinost, D., & Constable, R. T. (2020a). There is no single functional atlas even for a single individual: Functional parcel definitions change with task. NeuroImage, 208, 116366. 10.1016/j.neuroimage.2019.116366, 31740342

[bib207] Salehi, M., Karbasi, A., Barron, D. S., Scheinost, D., & Constable, R. T. (2020b). Individualized functional networks reconfigure with cognitive state. NeuroImage, 206, 116233. 10.1016/j.neuroimage.2019.116233, 31574322 PMC7216521

[bib208] Salimi-Khorshidi, G., Douaud, G., Beckmann, C. F., Glasser, M. F., Griffanti, L., & Smith, S. M. (2014). Automatic denoising of functional MRI data: Combining independent component analysis and hierarchical fusion of classifiers. NeuroImage, 90, 449–468. 10.1016/j.neuroimage.2013.11.046, 24389422 PMC4019210

[bib209] Salman, M. S., Wager, T. D., Damaraju, E., Abrol, A., Vergara, V. M., Fu, Z., & Calhoun, V. D. (2022). An approach to automatically label and order brain activity/component maps. Brain Connectivity, 12(1), 85–95. 10.1089/brain.2020.0950, 34039009 PMC8867103

[bib210] Salvo, J. J., Holubecki, A. M., & Braga, R. M. (2021). Correspondence between functional connectivity and task-related activity patterns within the individual. Current Opinion in Behavioral Sciences, 40, 178–188. 10.1016/j.cobeha.2021.05.003

[bib211] Sanchez, C. E., Richards, J. E., & Almli, C. R. (2012). Age-specific MRI templates for pediatric neuroimaging. Developmental Neuropsychology, 37(5), 379–399. 10.1080/87565641.2012.688900, 22799759 PMC3399736

[bib212] Sanchez-Romero, R., & Cole, M. W. (2021). Combining multiple functional connectivity methods to improve causal inferences. Journal of Cognitive Neuroscience, 33(2), 180–194. 10.1162/jocn_a_01580, 32427070 PMC8132338

[bib213] Sarwar, T., Tian, Y., Yeo, B. T. T., Ramamohanarao, K., & Zalesky, A. (2021). Structure-function coupling in the human connectome: A machine learning approach. NeuroImage, 226, 117609. 10.1016/j.neuroimage.2020.117609, 33271268

[bib214] Sassenberg, T. A, Burton, P. C., Mwilambwe-Tshilobo, L., Jung, R. E., Rustichini, A., Spreng, R. N., & DeYoung, C. G. (2023). Conscientiousness associated with efficiency of the salience/ventral attention network: Replication in three samples using individualized parcellation. NeuroImage, 272, 120081. 10.1016/j.neuroimage.2023.120081, 37011715 PMC10132286

[bib215] Satterthwaite, T. D., Wolf, D. H., Loughead, J., Ruparel, K., Elliott, M. A., Hakonarson, H., Gur, R. C., & Gur, R. E. (2012). Impact of in-scanner head motion on multiple measures of functional connectivity: Relevance for studies of neurodevelopment in youth. NeuroImage, 60(1), 623–632. 10.1016/j.neuroimage.2011.12.063, 22233733 PMC3746318

[bib216] Seeley, W. W., Menon, V., Schatzberg, A. F., Keller, J., Glover, G. H., Kenna, H., Reiss, A. L., & Greicius, M. D. (2007). Dissociable intrinsic connectivity networks for salience processing and executive control. Journal of Neuroscience, 27(9), 2349–2356. 10.1523/JNEUROSCI.5587-06.2007, 17329432 PMC2680293

[bib217] Seitzman, B. A., Gratton, C., Laumann, T. O., Gordon, E. M., Adeyemo, B., Dworetsky, A., Kraus, B. T., Gilmore, A. W., Berg, J. J., Ortega, M., Nguyen, A., Greene, D. J., McDermott, K. B., Nelson, S. M., Lessov-Schlaggar, C. N., Schlaggar, B. L., Dosenbach, N. U. F., & Petersen, S. E. (2019). Trait-like variants in human functional brain networks. Proceedings of the National Academy of Sciences of the United States of America, 116(45), 22851–22861. 10.1073/pnas.1902932116, 31611415 PMC6842602

[bib218] Setton, R., Mwilambwe-Tshilobo, L., Girn, M., Lockrow, A. W., Baracchini, G., Hughes, C., Lowe, A. J., Cassidy, B. N., Li, J., Luh, W.-M., Bzdok, D., Leahy, R. M., Ge, T., Margulies, D. S., Misic, B., Bernhardt, B. C., Stevens, W. D., De Brigard, F., Kundu, P., … Spreng, R. N. (2023). Age differences in the functional architecture of the human brain. Cerebral Cortex, 33(1), 114–134. 10.1093/cercor/bhac056, 35231927 PMC9758585

[bib219] Shafiei, G., Markello, R. D., Vos de Wael, R., Bernhardt, B. C., Fulcher, B. D., & Misic, B. (2020). Topographic gradients of intrinsic dynamics across neocortex. eLife, 9, e62116. 10.7554/eLife.62116, 33331819 PMC7771969

[bib220] Shine, J. M., Bissett, P. G., Bell, P. T., Koyejo, O., Balsters, J. H., Gorgolewski, K. J., Moodie, C. A., & Poldrack, R. A. (2016). The dynamics of functional brain networks: Integrated network states during cognitive task performance. Neuron, 92(2), 544–554. 10.1016/j.neuron.2016.09.018, 27693256 PMC5073034

[bib221] Shulman, G. L., Fiez, J. A., Corbetta, M., Buckner, R. L., Miezin, F. M., Raichle, M. E., & Petersen, S. E. (1997). Common blood flow changes across visual tasks: II. Decreases in cerebral cortex. Journal of Cognitive Neuroscience, 9(5), 648–663. 10.1162/jocn.1997.9.5.648, 23965122

[bib222] Sinke, M. R. T., Otte, W. M., Christiaens, D., Schmitt, O., Leemans, A., van der Toorn, A., Sarabdjitsingh, R. A., Joëls, M., & Dijkhuizen, R. M. (2018). Diffusion MRI-based cortical connectome reconstruction: Dependency on tractography procedures and neuroanatomical characteristics. Brain Structure & Function, 223(5), 2269–2285. 10.1007/s00429-018-1628-y, 29464318 PMC5968063

[bib223] Smith, D. M., Perez, D. C., Porter, A., Dworetsky, A., & Gratton, C. (2021). Light through the fog: Using precision fMRI data to disentangle the neural substrates of cognitive control. Current Opinion in Behavioral Sciences, 40, 19–26. 10.1016/j.cobeha.2020.12.004, 33553511 PMC7861476

[bib224] Smith, S. M., Beckmann, C. F., Andersson, J., Auerbach, E. J., Bijsterbosch, J., Douaud, G., Duff, E., Feinberg, D. A., Griffanti, L., Harms, M. P., Kelly, M., Laumann, T., Miller, K. L., Moeller, S., Petersen, S., Power, J., Salimi-Khorshidi, G., Snyder, A. Z., Vu, A. T., … WU-Minn HCP Consortium. (2013). Resting-state fMRI in the Human Connectome Project. NeuroImage, 80, 144–168. 10.1016/j.neuroimage.2013.05.039, 23702415 PMC3720828

[bib225] Smith, S. M., Fox, P. T., Miller, K. L., Glahn, D. C., Fox, P. M., Mackay, C. E., Filippini, N., Watkins, K. E., Toro, R., Laird, A. R., & Beckmann, C. F. (2009). Correspondence of the brain’s functional architecture during activation and rest. Proceedings of the National Academy of Sciences of the United States of America, 106(31), 13040–13045. 10.1073/pnas.0905267106, 19620724 PMC2722273

[bib226] Spadone, S., Della Penna, S., Sestieri, C., Betti, V., Tosoni, A., Perrucci, M. G., Romani, G. L., & Corbetta, M. (2015). Dynamic reorganization of human resting-state networks during visuospatial attention. Proceedings of the National Academy of Sciences of the United States of America, 112(26), 8112–8117. 10.1073/pnas.1415439112, 26080395 PMC4491799

[bib227] Sporns, O., Faskowitz, J., Teixeira, A. S., Cutts, S. A., & Betzel, R. F. (2021). Dynamic expression of brain functional systems disclosed by fine-scale analysis of edge time series. Network Neuroscience, 5(2), 405–433. 10.1162/netn_a_00182, 34189371 PMC8233118

[bib228] Spreng, R. N. (2022). OHBM Workgroup for HArmonized Taxonomy of NETworks (WHATNET). Open Science Framework. 10.17605/OSF.IO/3FZTA

[bib229] Spreng, R. N., Sepulcre, J., Turner, G. R., Stevens, W. D., & Schacter, D. L. (2013). Intrinsic architecture underlying the relations among the default, dorsal attention, and frontoparietal control networks of the human brain. Journal of Cognitive Neuroscience, 25(1), 74–86. 10.1162/jocn_a_00281, 22905821 PMC3816715

[bib230] Spronk, M., Keane, B. P., Ito, T., Kulkarni, K., Ji, J. L., Anticevic, A., & Cole, M. W. (2021). A whole-brain and cross-diagnostic perspective on functional brain network dysfunction. Cerebral Cortex, 31(1), 547–561. 10.1093/cercor/bhaa242, 32909037 PMC7947178

[bib231] Srirangarajan, T., Mortazavi, L., Bortolini, T., Moll, J., & Knutson, B. (2021). Multi-band fMRI compromises detection of mesolimbic reward responses. NeuroImage, 244, 118617. 10.1016/j.neuroimage.2021.118617, 34600102 PMC8626533

[bib232] Stanley, M. L., Gessell, B., & De Brigard, F. (2019). Network modularity as a foundation for neural reuse. Philosophy of Science, 86(1), 23–46. 10.1086/701037

[bib233] Stevens, W. D., & Spreng, R. N. (2014). Resting-state functional connectivity MRI reveals active processes central to cognition. Wiley Interdisciplinary Reviews: Cognitive Science, 5(2), 233–245. 10.1002/wcs.1275, 26304310

[bib234] Stevens, W. D., Tessler, M. H., Peng, C. S., & Martin, A. (2015). Functional connectivity constrains the category-related organization of human ventral occipitotemporal cortex. Human Brain Mapping, 36(6), 2187–2206. 10.1002/hbm.22764, 25704493 PMC4414790

[bib235] Suárez, L. E., Markello, R. D., Betzel, R. F., & Misic, B. (2020). Linking structure and function in macroscale brain networks. Trends in Cognitive Sciences, 24(4), 302–315. 10.1016/j.tics.2020.01.008, 32160567

[bib236] Sun, H., Yue, Q., Sy, J. L., Godwin, D., Eaton, H. P., Raghavan, P., & Marois, R. (2020). Increase in internetwork functional connectivity in the human brain with attention capture. Journal of Neurophysiology, 124(6), 1885–1899. 10.1152/jn.00693.2019, 33052763 PMC7814904

[bib237] Supekar, K., Uddin, L. Q., Prater, K., Amin, H., Greicius, M. D., & Menon, V. (2010). Development of functional and structural connectivity within the default mode network in young children. NeuroImage, 52(1), 290–301. 10.1016/j.neuroimage.2010.04.009, 20385244 PMC2976600

[bib238] Sylvester, C. M., Yu, Q., Srivastava, A. B., Marek, S., Zheng, A., Alexopoulos, D., Smyser, C. D., Shimony, J. S., Ortega, M., Dierker, D. L., Patel, G. H., Nelson, S. M., Gilmore, A. W., McDermott, K. B., Berg, J. J., Drysdale, A. T., Perino, M. T., Snyder, A. Z., Raut, R. V., … Dosenbach, N. U. F. (2020). Individual-specific functional connectivity of the amygdala: A substrate for precision psychiatry. Proceedings of the National Academy of Sciences of the United States of America, 117(7), 3808–3818. 10.1073/pnas.1910842117, 32015137 PMC7035483

[bib239] Thomsen, K., Offenhauser, N., & Lauritzen, M. (2004). Principal neuron spiking: Neither necessary nor sufficient for cerebral blood flow in rat cerebellum. Journal of Physiology, 560(1), 181–189. 10.1113/jphysiol.2004.068072, 15272036 PMC1665203

[bib240] Thomsen, K., Piilgaard, H., Gjedde, A., Bonvento, G., & Lauritzen, M. (2009). Principal cell spiking, postsynaptic excitation, and oxygen consumption in the rat cerebellar cortex. Journal of Neurophysiology, 102(3), 1503–1512. 10.1152/jn.00289.2009, 19571198

[bib241] Uddin, L. Q. (2013). Complex relationships between structural and functional brain connectivity. Trends in Cognitive Sciences, 17(12), 600–602. 10.1016/j.tics.2013.09.011, 24094797 PMC3858496

[bib242] Uddin, L. Q., Kelly, A. M., Biswal, B. B., Castellanos, F. X., & Milham, M. P. (2009). Functional connectivity of default mode network components: correlation, anticorrelation, and causality. Human Brain Mapping, 30(2), 625–637. 10.1002/hbm.20531, 18219617 PMC3654104

[bib243] Uddin, L. Q., Kelly, A. M. C., Biswal, B. B., Margulies, D. S., Shehzad, Z., Shaw, D., Ghaffari, M., Rotrosen, J., Adler, L. A., Castellanos, F. X., & Milham, M. P. (2008a). Network homogeneity reveals decreased integrity of default-mode network in ADHD. Journal of Neuroscience Methods, 169(1), 249–254. 10.1016/j.jneumeth.2007.11.031, 18190970

[bib244] Uddin, L. Q., Mooshagian, E., Zaidel, E., Scheres, A., Margulies, D. S., Kelly, A. M., Shehzad, Z., Adelstein, J. S., Castellanos, F. X., Biswal, B. B., & Milham, M. P. (2008b). Residual functional connectivity in the split-brain revealed with resting-state functional MRI. Neuroreport, 19(7), 703–709. 10.1097/WNR.0b013e3282fb8203, 18418243 PMC3640406

[bib245] Uddin, L. Q., Supekar, K. S., Ryali, S., & Menon, V. (2011). Dynamic reconfiguration of structural and functional connectivity across core neurocognitive brain networks with development. Journal of Neuroscience, 31(50), 18578–18589. 10.1523/JNEUROSCI.4465-11.2011, 22171056 PMC3641286

[bib246] Uddin, L. Q., Yeo, B. T. T., & Spreng, R. N. (2019). Towards a universal taxonomy of macro-scale functional human brain networks. Brain Topography, 32(6), 926–942. 10.1007/s10548-019-00744-6, 31707621 PMC7325607

[bib247] Vaishnavi, S. N., Vlassenko, A. G., Rundle, M. M., Snyder, A. Z., Mintun, M. A., & Raichle, M. E. (2010). Regional aerobic glycolysis in the human brain. Proceedings of the National Academy of Sciences of the United States of America, 107(41), 17757–17762. 10.1073/pnas.1010459107, 20837536 PMC2955101

[bib248] Van Dijk, K. R. A., Sabuncu, M. R., & Buckner, R. L. (2012). The influence of head motion on intrinsic functional connectivity MRI. NeuroImage, 59(1), 431–438. 10.1016/j.neuroimage.2011.07.044, 21810475 PMC3683830

[bib249] Van Essen, D. C., & Glasser, M. F. (2018). Parcellating cerebral cortex: How invasive animal studies inform noninvasive mapmaking in humans. Neuron, 99(4), 640–663. 10.1016/j.neuron.2018.07.002, 30138588 PMC6149530

[bib250] Van Essen, D. C., Smith, S. M., Barch, D. M., Behrens, T. E. J., Yacoub, E., Ugurbil, K., & WU-Minn HCP Consortium. (2013). The WU-Minn Human Connectome Project: An overview. NeuroImage, 80, 62–79. 10.1016/j.neuroimage.2013.05.041, 23684880 PMC3724347

[bib251] Vázquez-Rodríguez, B., Suarez, L. E., Markello, R. D., Shafiei, G., Paquola, C., Hagmann, P., van den Heuvel, M. P., Bernhardt, B. C., Spreng, R. N., & Misic, B. (2019). Gradients of structure-function tethering across neocortex. Proceedings of the National Academy of Sciences of the United States of America, 116(42), 21219–21227. 10.1073/pnas.1903403116, 31570622 PMC6800358

[bib252] Varela, F., Lachaux, J.-P., Rodriguez, E., & Martinerie, J. (2001). The brainweb: Phase synchronization and large-scale integration. Nature Reviews Neuroscience, 2(4), 229–239. 10.1038/35067550, 11283746

[bib253] Veit, M. J., Kucyi, A., Hu, W., Zhang, C., Zhao, B., Guo, Z., Yang, B., Sava-Segal, C., Perry, C., Zhang, J., Zhang, K., & Parvizi, J. (2021). Temporal order of signal propagation within and across intrinsic brain networks. Proceedings of the National Academy of Sciences of the United States of America, 118(48), e2105031118. 10.1073/pnas.2105031118, 34819365 PMC8640784

[bib254] Vij, S. G., Nomi, J. S., Dajani, D. R., & Uddin, L. Q. (2018). Evolution of spatial and temporal features of functional brain networks across the lifespan. NeuroImage, 173, 498–508. 10.1016/j.neuroimage.2018.02.066, 29518568 PMC6613816

[bib255] Vincent, J. L., Kahn, I., Snyder, A. Z., Raichle, M. E., & Buckner, R. L. (2008). Evidence for a frontoparietal control system revealed by intrinsic functional connectivity. Journal of Neurophysiology, 100(6), 3328–3342. 10.1152/jn.90355.2008, 18799601 PMC2604839

[bib256] Wang, D., Buckner, R. L., Fox, M. D., Holt, D. J., Holmes, A. J., Stoecklein, S., Langs, G., Pan, R., Qian, T., Li, K., Baker, J. T., Stufflebeam, S. M., Wang, K., Wang, X., Hong, B., & Liu, H. (2015). Parcellating cortical functional networks in individuals. Nature Neuroscience, 18(12), 1853–1860. 10.1038/nn.4164, 26551545 PMC4661084

[bib257] Wang, S., Tepfer, L. J., Taren, A. A., & Smith, D. V. (2020). Functional parcellation of the default mode network: A large-scale meta-analysis. Scientific Reports, 10(1), 16096. 10.1038/s41598-020-72317-8, 32999307 PMC7528067

[bib258] Whitfield-Gabrieli, S., Thermenos, H. W., Milanovic, S., Tsuang, M. T., Faraone, S. V., McCarley, R. W., Shenton, M. E., Green, A. I., Nieto-Castanon, A., LaViolette, P., Wojcik, J., Gabrieli, J. D. E., & Seidman, L. J. (2009). Hyperactivity and hyperconnectivity of the default network in schizophrenia and in first-degree relatives of persons with schizophrenia. Proceedings of the National Academy of Sciences of the United States of America, 106(4), 1279–1284. 10.1073/pnas.0809141106, 19164577 PMC2633557

[bib259] Wig, G. S. (2017). Segregated systems of human brain networks. Trends in Cognitive Sciences, 21(12), 981–996. 10.1016/j.tics.2017.09.006, 29100737

[bib260] Wig, G. S., Laumann, T. O., & Petersen, S. E. (2014). An approach for parcellating human cortical areas using resting-state correlations. NeuroImage, 93(Pt 2), 276–291. 10.1016/j.neuroimage.2013.07.035, 23876247 PMC3912214

[bib261] Wirsich, J., Jorge, J., Iannotti, G. R., Shamshiri, E. A., Grouiller, F., Abreu, R., Lazeyras, F., Giraud, A.-L., Gruetter, R., Sadaghiani, S., & Vulliémoz, S. (2021). The relationship between EEG and fMRI connectomes is reproducible across simultaneous EEG-fMRI studies from 1.5T to 7T. NeuroImage, 231, 117864. 10.1016/j.neuroimage.2021.117864, 33592241

[bib262] Wirsich, J., Ridley, B., Besson, P., Jirsa, V., Bénar, C., Ranjeva, J.-P., & Guye, M. (2017). Complementary contributions of concurrent EEG and fMRI connectivity for predicting structural connectivity. NeuroImage, 161, 251–260. 10.1016/j.neuroimage.2017.08.055, 28842386

[bib263] Xue, A., Kong, R., Yang, Q., Eldaief, M. C., Angeli, P. A., DiNicola, L. M., Braga, R. M., Buckner, R. L., & Yeo, B. T. T. (2021). The detailed organization of the human cerebellum estimated by intrinsic functional connectivity within the individual. Journal of Neurophysiology, 125(2), 358–384. 10.1152/jn.00561.2020, 33427596 PMC7948146

[bib264] Yendiki, A., Aggarwal, M., Axer, M., Howard, A. F. D., van Cappellen van Walsum, A.-M., & Haber, S. N. (2022). Post mortem mapping of connectional anatomy for the validation of diffusion MRI. NeuroImage, 256, 119146. 10.1016/j.neuroimage.2022.119146, 35346838 PMC9832921

[bib265] Yeo, B. T. T., Krienen, F. M., Chee, M. W. L., & Buckner, R. L. (2014). Estimates of segregation and overlap of functional connectivity networks in the human cerebral cortex. NeuroImage, 88, 212–227. 10.1016/j.neuroimage.2013.10.046, 24185018 PMC4007373

[bib266] Yeo, B. T. T., Krienen, F. M., Sepulcre, J., Sabuncu, M. R., Lashkari, D., Hollinshead, M., Roffman, J. L., Smoller, J. W., Zöllei, L., Polimeni, J. R., Fischl, B., Liu, H., & Buckner, R. L. (2011). The organization of the human cerebral cortex estimated by intrinsic functional connectivity. Journal of Neurophysiology, 106(3), 1125–1165. 10.1152/jn.00338.2011, 21653723 PMC3174820

[bib267] Yuan, R., Biswal, B. B., & Zaborszky, L. (2019). Functional subdivisions of magnocellular cell groups in human basal forebrain: Test-retest resting-state study at ultra-high field, and meta-analysis. Cerebral Cortex, 29(7), 2844–2858. 10.1093/cercor/bhy150, 30137295 PMC6611453

[bib268] Yuste, R. (2015). From the neuron doctrine to neural networks. Nature Reviews Neuroscience, 16(8), 487–497. 10.1038/nrn3962, 26152865

[bib269] Zalesky, A., Fornito, A., & Bullmore, E. (2012). On the use of correlation as a measure of network connectivity. NeuroImage, 60(4), 2096–2106. 10.1016/j.neuroimage.2012.02.001, 22343126

[bib270] Zalesky, A., Fornito, A., Cocchi, L., Gollo, L. L., & Breakspear, M. (2014). Time-resolved resting-state brain networks. Proceedings of the National Academy of Sciences of the United States of America, 111(28), 10341–10346. 10.1073/pnas.1400181111, 24982140 PMC4104861

[bib271] Zhang, J., Kucyi, A., Raya, J., Nielsen, A. N., Nomi, J. S., Damoiseaux, J. S., Greene, D. J., Horovitz, S. G., Uddin, L. Q., & Whitfield-Gabrieli, S. (2021). What have we really learned from functional connectivity in clinical populations? NeuroImage, 242, 118466. 10.1016/j.neuroimage.2021.118466, 34389443

[bib272] Zheng, A., Montez, D. F., Marek, S., Gilmore, A. W., Newbold, D. J., Laumann, T. O., Kay, B. P., Seider, N. A., Van, A. N., Hampton, J. M., Alexopoulos, D., Schlaggar, B. L., Sylvester, C. M., Greene, D. J., Shimony, J. S., Nelson, S. M., Wig, G. S., Gratton, C., McDermott, K. B., … Dosenbach, N. U. F. (2021). Parallel hippocampal-parietal circuits for self- and goal-oriented processing. Proceedings of the National Academy of Sciences of the United States of America, 118(34), e2101743118. 10.1073/pnas.2101743118, 34404728 PMC8403906

[bib273] Zhong, S., He, Y., & Gong, G. (2015). Convergence and divergence across construction methods for human brain white matter networks: An assessment based on individual differences. Human Brain Mapping, 36(5), 1995–2013. 10.1002/hbm.22751, 25641208 PMC6869604

